# Lithium rescues cultured rat metatarsals from dexamethasone-induced growth failure

**DOI:** 10.1038/s41390-024-03192-6

**Published:** 2024-04-29

**Authors:** Ondrej Soucek, Ondrej Cinek, Lilly Velentza, Valerij Semjonov, Martin Bezdicka, Farasat Zaman, Lars Sävendahl

**Affiliations:** 1 Vera Vavrova Lab/VIAL, Department of Paediatrics, Second Faculty of Medicine, Charles University and Motol University Hospital, Prague, Czech Republic; 2 Paediatric Endocrinology Unit, Department of Children’s and Women’s Health, Karolinska Institutet, Stockholm, Sweden; 3 Department of Paediatrics and Department of Medical Microbiology, Second Faculty of Medicine, Charles University and Motol University Hospital, Prague, Czech Republic; 4 Astrid Lindgren Children´s Hospital, Karolinska University Hospital, Stockholm, Sweden

## Abstract

**Background:**

Glucocorticoids are commonly used in children with different chronic diseases. Growth failure represents a so far untreatable undesired side-effect. As lithium chloride (LiCl) is known to induce cell renewal in various tissues, we hypothesized that LiCl may prevent glucocorticoid-induced growth failure.

**Methods:**

We monitored growth of fetal rat metatarsals cultured ex-vivo with dexamethasone and/or LiCl, while molecular mechanisms were explored through RNA sequencing by implementing the differential gene expression and gene set analysis. Quantification of β-catenin in human growth plate cartilage cultured with dexamethasone and/or LiCl was added for verification.

**Results:**

After 14 days of culture, the length of dexamethasone-treated fetal rat metatarsals increased by 1.4 ± 0.2 mm compared to 2.4 ± 0.3 mm in control bones (*p* < 0.001). The combination of LiCl and dexamethasone led to bone length increase of 1.9 ± 0.3 mm (*p* < 0.001 vs. dexamethasone alone). By adding lithium, genes for cell cycle and Wnt/β-catenin, Hedgehog and Notch signaling, were upregulated compared to dexamethasone alone group.

**Conclusions:**

LiCl has the potential to partially rescue from dexamethasone-induced bone growth impairment in an ex vivo model. Transcriptomics identified cell renewal and proliferation as candidates for the underlying mechanisms. Our observations may open up the development of a new treatment strategy for bone growth disorders.

**Impact:**

- LiCl is capable to prevent glucocorticoid-induced growth failure in rat metatarsals in vitro.
- The accompanying drug-induced transcriptomic changes suggested cell renewal and proliferation as candidate underlying mechanisms.
- Wnt/beta-catenin pathway could be one of those novel mechanisms.

## Introduction

Glucocorticoids (GCs) have both anti-inflammatory and immunosuppressive effects and are commonly used in many different pediatric diseases, including asthma, nephrotic syndrome, inflammatory bowel disease, Duchenne muscle dystrophy and acute leukemia.^1^ However, their use often results in undesired side-effects involving the skeletal system, such as osteoporosis and suppression of longitudinal bone growth in children.^2,3^ Besides the well-described systemic growth-inhibiting effects mediated mainly through the suppression of the growth hormone/insulin-like growth factor 1 axis,^4^ many studies have reported the direct adverse effects of GCs in the growth plate cartilage, the main site of linear bone growth. It has been previously reported that one of the mechanisms governing these local side-effects of GCs is the induction of apoptosis in growth plate chondrocytes,^5^ through an increase in the expression of the pro-apoptotic protein Bax.^6^

Interestingly, exogenous GCs have been also shown to impair osteoblast and chondrocyte differentiation via downregulating the Wnt/β-catenin pathway, another key signaling cascade implicated in bone development and local growth plate regulation.^7,8^ Wingless-related integration site proteins (Wnt:s) belong to a family of secreted cysteine-rich glycoproteins which signal via the Wnt/β-catenin pathway by binding to the membrane receptor complex.^9^ Glycogen synthase kinase 3 beta (GSK3β) represents one of the intracellular modulators of the Wnt/β-catenin pathway. GSK3β is an active compound of a larger molecular complex that phosphorylates β-catenin and thus causes its degradation. Stimulation of GSK3β leads to inhibition of Wnt/β-catenin signaling, whereas the inhibition causes increased (non-phosphorylated) β-catenin concentration and its translocation into the nucleus, where it increases target gene transcription.

Lithium chloride (LiCl) is a known GSK3β inhibitor and the treatment with LiCl increases the proliferation of human mesenchymal stem cells^10^ and also rescues from glucocorticoid-induced apoptosis of spontaneously immortalized murine calvarial osteoblasts.^11^ Lithium has been used as a mood stabilizer in clinical practice for decades,^12^ its neuroprotection in psychotic patients was documented^13^ and is also used in pediatric patients to treat bipolar disorder.^14^

Based on the importance of Wnt signaling in bone development and the properties of LiCl as a Wnt modulator, we here aimed to investigate the potential of LiCl to rescue from growth failure caused by a clinically used glucocorticoid, dexamethasone, while trying to explore the molecular mechanisms behind the effects of the two drugs. In this study we used a well-established ex vivo model of cultured fetal rat metatarsal bones where growth can be tracked in a real-time manner^15,16^ and a unique model of cultured human growth plate cartilage.^17^ We utilized RNA sequencing (RNA-seq) in rat metatarsal tissue to comprehensively explore molecular pathways that govern the observed effects of dexamethasone and LiCl on longitudinal bone growth.

## Material and methods

### Fetal rat metatarsal bone cultures

The pregnant Sprague-Dawley rats (day 19 after mating/fertilization) were euthanized with CO_2_, and the fetuses were excised and euthanized by decapitation. The paws were collected in sterile medium (DMEM/F12 with 40 μM Gentamicin and 2.7 μM Fungizone) and transferred on ice. The three middle metatarsal bones were dissected from the fetuses’ hind paws as described earlier.^17^ Immediately after dissection, metatarsal bones were kept in the incubator (37 °C, 5.0% CO_2_) in the culture medium (DMEM/F12 with 40 μM Gentamicin, 0.2% Bovine serum albumin, 1 mM β-Glycerolphosphate and 285 μM Ascorbic acid). Each bone was then transferred in a separate well of the 24-well plate containing 1.0 mL culture media.

In the first experiments, we established the dosing of lithium chloride: metatarsal bones were randomly distributed into 8 treatment groups: Control, Dexamethasone (1 μM), LiCl (0.1 mM), LiCl (1 mM), LiCl (10 mM) and both drugs combined (all three LiCl concentrations).

Based on the observed dose-dependent effect of LiCl, subsequent experiments were designed with four treatment groups: (1) Control, (2) Dexamethasone only (1 μM), (3) LiCl only (10 mM) and (4) Dexamethasone (1 μM) + LiCl (10 mM). Culture media was changed every 2-3 days and digital pictures were taken (a microscope with CCD camera) at days 0, 2, 5, 7, 9, 12, and 14 (termination of culture). The length of bones was measured from captured pictures by using ImageJ software (freely available NIH project).^18^ The length of each bone at each time point was measured, and the increase in length from day 0 was calculated. Each bone was cultured separately and regarded as an independent observation as previously detailed.^17^ As three rats were processed for the growth study, mixed effects model was first used to test the random intercept related to the source of the bone. The variability proportion of bone length increase denoted to rat source was only 4.2% at day 14 (the endpoint). Hence, we conducted our analysis using linear regression model. The *p*-values for group differences were obtained from the linear model with bone length increase from day 0 at days 2, 5, 7, 9, 12 and 14, respectively, as a response and treatment group as an explanatory variable. *P*-values were adjusted using FDR correction.

Three additional consequent experiments on freshly dissected metatarsals served for gene expression analysis: RNA was extracted from the bones after 72 h of culture (random distribution of 20 bones/treatment group/experiment).

### RNA extraction and sequencing

The metatarsal bones of each treatment group were combined and the TRIzol reagent protocol (Life Technologies, #15596-026) was used for RNA extraction (0.5 mL of Trizol per sample, i.e. per treatment group). The tissue homogenization (2 × 45 s with Speed 2) was performed by using the bead-beating technology (Minilys device, Precellys Lysing kit MK28-R, Bertin). Chloroform (Sigma Aldrich, #C2432-25mL), 70%-ethanol and spin columns with ready-to-use buffers and clean water (Thermo Fisher Scientific, PureLink RNA Mini Kit, #12183018A) were used to extract RNA. The RNA concentration and purity were assessed with a spectrophotometer (Thermo Fisher Scientific, NanoDrop One^C^). The RNA yield ranged between 26–94 ng/µL and purity (A260/A280 ratio) was 1.6–1.9.

The RNAseq libraries were prepared as follows: 300 ng of total RNA was subjected to library synthesis using the SureSelect strand-specific mRNA library preparation kit (protocol version E0). The RNA was enriched for the Poly-A mRNA and reverse transcribed to double-stranded cDNA. The cDNA was end-repaired, adenylated, and ligated with Illumina indexes prior to 14 cycles of PCR. The final concentration of prepared RNAseq libraries was 2.7–3.0 pM. Sequencing was performed on Illumina NextSeq 500 instrument using the High Output kit (Illumina) and 2 × 75 paired-end cycles.

### Differential gene expression and pathway analysis

The transcriptome profiles were analyzed using the BRB-SeqTools framework (https://brb.nci.nih.gov/seqtools/): reads were aligned using Subread, duplicated reads eliminated by Picard, and genes counted by HTSeq-count.^19^ The gene counts tables and the sample metadata were assembled into a DESeq2 DESeqDataSet object,^20^ and mutual distances of technical replicates were inspected in a PCA plot. Upon verification of their similarity, technical replicates were merged, which resulted in libraries having 9.0–21.9 million reads. Genes were filtered for having 60 reads or more in at least 2 different samples - 12,592 genes were retained. Global differences in gene expression between the four conditions were assessed on ordination plots, and particular differentially expressed genes sought for using a negative binomial regression model in DESeq2. There were four conditions in a factorial design which was reflected in the analyzed contrasts. Results were visualized using the MA-plots, volcano plots, heatmaps and count plots of the genes of interest. The DESeq test *P* values were corrected for multiple testing using Benjamini–Hochberg method. A minimum of 2-fold increase or decrease in expression, and a corrected *P* value of <0.05 was needed for a gene to be considered. Gene count tables and raw sequencing data were deposited at NCBI Gene Expression Omnibus (GEO), and Sequencing Reads Archive (SRA) databases under accession number GSE186104.

Web-based application Reactome (https://reactome.org) was used to perform gene set (pathway) analysis. Camera was selected as the analysis method (correlation adjusted mean rank), which is based on two-sided t-test allowing for correlation.^21^ The test statistic is modified by variance inflation factor estimated from the data. Hence, it takes into account inter-gene correlation. It is tested whether the fold-differences as a result of treatment with both dexamethasone and LiCl differ between the gene set of interest and the background. Dexamethasone alone group is taken as a reference group. FDR approach is applied to adjust for multiple testing procedure. The threshold for FDR- adjusted *P*-value was set to 0.05.

### Immunohistochemistry of metatarsals

A limited number of spare metatarsals that were left from the first two growth experiments were used for immunohistochemistry staining (i.e., 1 metatarsal/treatment group/rat). Paraffin sections of fetal rat metatarsal bones (5 μm) were deparaffinized and rehydrated in graded ethanols. Next, antigen retrieval was performed with citrate buffer (10 mM, pH 6.0 at 75 °C for 15 min) or trypsin solution (for the activated β-catenin antibody; 37 °C for 10 min), followed by blocking with 2% goat serum for 1 h. The slides were incubated overnight with the primary antibody at 4 °C. The following antibodies were used: Proliferative cell nuclear antigen (PCNA; 1:2000; Abcam Cat# ab18197, RRID: AB_444313), B-cell lymphoma 2 antigen (BCL-2; 1:50; Abcam Cat# ab196495, RRID: AB_2924862), Non-phospho (Active) β-Catenin (Ser33/37/Thr41) (D13A1) (1:50; Cell Signaling Technology Cat# 8814, RRID:AB_11127203). The secondary antibodies (Abcam Cat# ab97049, RRID: AB_10679577 or Abcam Cat# ab6788, RRID: AB_954885) were added for 1 h, followed by incubation with horseradish peroxidase (HRP)-conjugated streptavidin (VECTASTAIN® ABC-HRP Kit, VECTOR Laboratories and 3,3’ diaminobenzidine (DAB) development (Dako K3468). Alcian blue solution was used as counterstain. The plugin Immunohistochemistry Image Analysis Toolbox in Image J software (NIH, Bethesda, MD) was used to quantify each bone’s area of active-β-catenin staining in the total area of the metatrsal bone (in percent).

### Human growth plate tissue

Samples were collected from 4 constitutionally tall stature patients (2 boys and 2 girls, aged 11–14 years, pubertal stages 2–4 according to Tanner) during their epiphysiodesis surgery. A previously published protocol was used to handle human growth plate tissue.^22^ The cylinder specimens were transferred in the collection medium (DMEM with 25 mM HEPES, 4 mM Glutamine, 25 mM Glucose and 21 µM Gentamicin) on ice. Thin transverse slices (0.5–1.0 mm), cut under a dissection microscope in sterile conditions in a laminar flow box, were randomly sorted into 4 groups (4–5 slices per group) and placed in 2.0 mL culture media (collection medium with 285 µM Ascorbic acid, 1 mM β-Glycerolphosphate and 0.2% Bovine serum albumin) per group on a 24 well plate. The treatment groups included Control, Dexamethasone (1 μM) and LiCl (1 mM) alone or in combination. After 24 h of treatment in the incubator (37 °C, 5.0% CO_2_), medium was removed, growth plate slices were washed with 1X PBS and fixed with 4% paraformaldehyde for 24 h at 4 °C. The slices were then decalcified with 10% EDTA for 24 h at 4 °C and afterwards stored in 70% ethanol. Paraffin embedment was used to prepare glass-mounted histologic sections.

### Immunohistochemistry and signal intensity evaluation

After deparaffinization, sections were treated with active β-catenin rabbit mAb (Cell Signaling Technology, #8814). Cyanin 3-labeled (CY3), anti-rabbit secondary Ab produced in donkey (Jackson ImmunoResearch Laboratories, Inc., #711-165-152) was used for immunofluorescent visualization. Nuclei were counterstained with DAPI (Vector laboratories, #H-1200). Confocal microscope with fluorescent filters (Nikon ECLIPSE E8000) connected to CCD camera (Olympus DP 70) was used to gain digital pictures of the sections through 20× magnification lens (Nikon Plan Apo, DIC, M, ∞/0.17, WD 1.0). The images were processed by ImageJ software. The staining intensity was assessed by calculating the total β-catenin-positive area (μm^2^) normalized to the number of DAPI-stained nuclei within the same area of the growth plate. Two-sample two-tailed t-tests were used to compare the results between two groups (dexamethasone vs. control, lithum vs. control and dexamethasone + lithum vs. dexamethasone alone). FDR method was used for multiple comparison adjustment.

### Statistical analyses

All analyses were performed and all figures were created using the statistical computing environment R, version 3.6.1.^23^ The significance threshold was *p* = 0.05. Whenever boxplots were used, it summarized the median (line inside the box), first and third quartile (i.e. Q1 and Q3, respectively; lower and upper box margin), minimum and maximum (Q1–1.5 × IQR and Q3 + 1.5 × IQR, where IQR means interquartile range; whiskers) and, eventually, outliers (<Q1–1.5 × IQR or >Q3 + 1.5 × IQR).

### Ethical approvals

The animal experiments involving ex vivo culture of fetal rat metatarsal bones were approved by the local ethical committees (Permit No:N637/12, Stockholm North Animal Ethics Committee, and Permit No: MSMT-15719/2016-2, Ministry of Education Youth and Sports Ethics Committee, Prague). Three Rs principle (replacement, reduction, refinement) was followed accordingly.^24^ The collection of human growth plate tissue was approved by the local ethics committee (Permit No: 97–214, Karolinska Institutet Research Ethics Committee North at the Karolinska University Hospital, Stockholm, Sweden). Informed consent was obtained from each individual and their legal guardians according to the Helsinki Declaration and documented in the original hospital records.

## Results

### Effects of dexamethasone and LiCl on fetal rat metatarsal bone growth

**Fig. 1:**
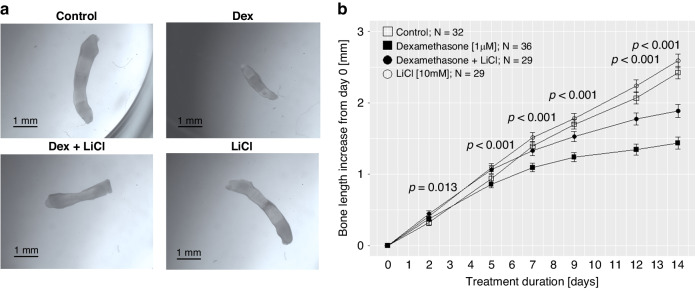
In vitro growth of fetal rat metatarsal bones in culture. Lithium chloride (LiCl) prevented dexamethasone-induced growth failure. **a** Representative images of metatarsal bones at day 14. **b** Growth curves of metatarsals over 14 days in culture for the four treatment groups. Means and their 95% confidence intervals were calculated from a linear regression model which explored the association between bone length increases (in mm) from day zero at day 2, 5, 7, 9, 12, and 14 (as response), respectively, and the four different treatment groups (as explanatory variable). The *p*-values correspond to the comparisons between Dexamethasone and Dexamethasone + LiCl groups and were adjusted using FDR correction. Dex dexamethasone, LiCl lithium chloride.

**Fig. 2:**
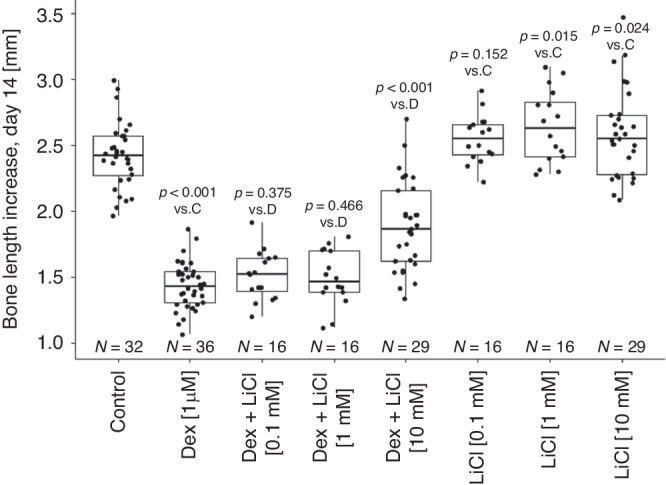
Fetal rat metatarsal bone length increase after 14 days of culture. The 10 mM concentration of LiCl prevented approx. 56% of the growth retardation caused by dexamethasone alone. Boxplots of treatment groups are shown with individual data points. *P*-values were obtained from linear model with bone length increase at day 14 as a response and treatment group as an explanatory variable. *P*-values were adjusted using FDR correction. Boxplots show the median (line inside the box), first and third quartile (i.e. Q1 and Q3, respectively; lower and upper box margin), minimum and maximum (Q1–1.5 × IQR and Q3 + 1.5 × IQR, where IQR means interquartile range; whiskers) and, eventually, outliers (<Q1–1.5 × IQR or >Q3 + 1.5 × IQR). Dex dexamethasone, LiCl lithium chloride.

Dexamethasone-treated metatarsals grew similar as control bones up to day 5 of culture. Thereafter, dexamethasone-treated bones grew less than control (Fig. 1). The bone length increased by 1.44 ± 0.18 mm (mean ± standard deviation) in the dexamethasone group and by 2.43 ± 0.25 mm in the control group during the 14 day-period of culture (Fig. 1). Bones treated with LiCl alone grew similarly as control (2.55 ± 0.18 mm, 2.64 ± 0.28 mm and 2.59 ± 0.33 mm increase, for 0.1 mM, 1.0 mM and 10 mM LiCl concentration, respectively; versus 2.43 ± 0.25 mm increase for control bones; Fig. 2). Interestingly, the highest LiCl concentration (10 mM) significantly prevented dexamethasone-induced growth retardation (1.89 ± 0.34 mm increase for dexamethasone + LiCl group versus 1.44 ± 0.18 mm increase in the dexamethasone group; Fig. 2). The growth rescuing effect was partial amounting to 56% (95% confidence interval 40–71%) of the dexamethasone-induced growth reduction. The addition of the two lower LiCl concentrations (0.1 mM and 1 mM) to dexamethasone did not rescue bone growth when compared to dexamethasone alone (1.51 ± 0.18 mm and 1.49 ± 0.21 mm increase in growth, respectively, versus 1.44 ± 0.18 mm in the dexamethasone group; Fig. 2).

### Effects of dexamethasone and LiCl on transcriptome of metatarsal bones

**Fig. 3:**
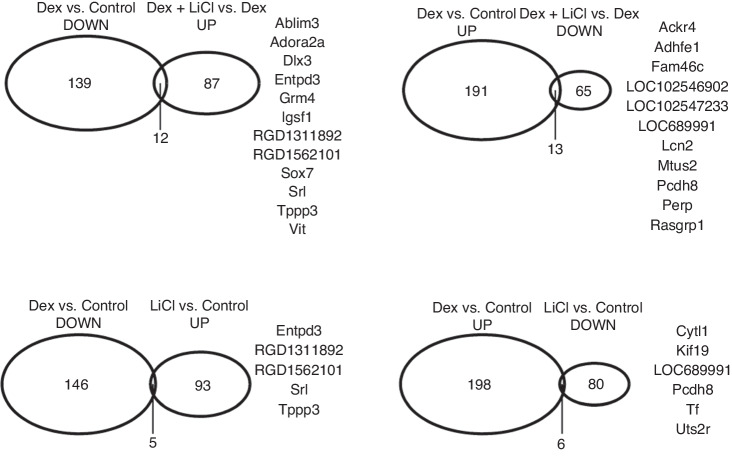
Venn diagrams illustrating the overlap of significantly differentially expressed genes in metatarsals. The number of overlapping genes is given in the intersection of the ellipses and they are listed on the side. Dex dexamethasone, LiCl lithium chloride.

**Fig. 4:**
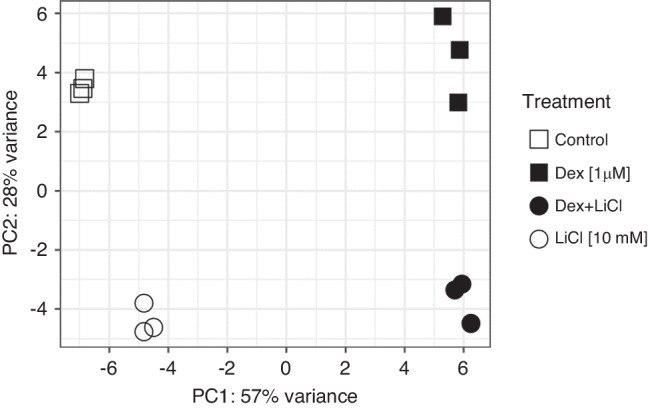
Principal component analysis plot showing the distribution of metatarsal transcriptome profiles. Each point represents transcriptome of a pool of metatarsals from one rat (and treatment group), three rats were used. The samples clustered according to the treatment group, thus within group variance was much less compared to intergroup variability. Dex dexamethasone, LiCl lithium chloride.

Transcriptome profiles of the cultured fetal rat metatarsal bones differed depending on which drug (or its combination) was added into the culture medium. A total of 355 genes were significantly affected by dexamethasone (fold difference >2.0 or <0.5 and false-discovery rate-corrected *P* < 0.05, Supplementary Table 1), of which 204 were up-regulated and 151 were down-regulated (the cross-tabulation of gene expression counts can be accessed at: https://www.ncbi.nlm.nih.gov/geo/query/acc.cgi?acc=GSE186104), as compared to control metatarsals. Lithium influenced the expression of 184 genes, of which 98 were up-regulated and 86 were down-regulated (Supplementary Table 2). Metatarsals treated with dexamethasone plus lithium together showed 177 differentially expressed genes when compared to the dexamethasone alone group (Supplementary Table 3) and 536 differentially expressed genes when compared to control group (Supplementary Table 4). The Supplementary Tables 1–4 list the genes along with the fold-differences and FDR-adjusted *p*-values for all the four comparisons of interest. The overlap of genes expressed in an opposite direction for particular comparisons of interest are presented on Venn diagrams in Fig. 3. These genes are involved in cell attachment (Ablim3, Pcdh8, Perp, Vit), cytokine production (RGD1311892, Cytl1), chemokine binding (Ackr4), G protein-coupled receptor signaling (Adora2a, Grm4, Ackr4), transcription activity (Dlx3, Sox7, Rasgrp1) or cell kinesis (Kif19, Srl, Tppp3, Vit, Mtus2). When looking at the whole transcriptome level, ordination (principal component analysis, PCA) showed an excellent clustering of the biological replicates of the transcriptomes into four clearly separated groups corresponding to the four distinct contents of the culture medium (Fig. 4). Thus, much higher variability was confirmed between the treatment groups than within single groups.

Gene set analysis showed that 55 pathways were significantly regulated in the dexamethasone + LiCl group versus dexamethasone alone group. Of those, 43 were upregulated (Table 1a) and 12 were downregulated (Table 1b). The upregulated pathways belonged to cell cycle domain (DNA damage control, synthesis and elongation), RNA synthesis and processing, cellular responses to chemical stress and metal ions, and respiratory electron transport (energy production). In addition, Wnt (cell proliferation, fate decision and stem cell maintenance; of which β-catenin is a target molecule), Hedgehog (limb bud formation and neural tube patterning) and Notch4 (involved in Wnt signaling and adipocyte differentiation) pathways were among the significantly upregulated gene sets. In contrast, transcription (RUNX1-induced B-lymphocytes differentiation, interleukin signaling and cell migration), cellular junction organization or hemostasis and complement cascade activation were downregulated (for enrichment plots se Supplementary Table 5). All pathways significantly regulated in at least one of the three two-group comparisons (i.e., dexamethasone vs. control, LiCl vs. control, and dexamethasone + LiCl vs. dexamethasone) are listed in Supplementary Table 6. There was no overlap of pathways except the Branch-chained amino acid catabolism which was upregulated by dexamethasone vs. control and downregulated by dexamethasone + LiCl vs. dexamethasone alone group.

**Table 1:** a. Gene sets (pathways) found to be significantly UP-regulated in dexamethasone + lithium group compared to dexamethasone alone group. b. Gene sets (pathways) found to be significantly DOWN-regulated in dexamethasone + lithium group compared to dexamethasone alone group.

a		
Pathway Name	Cellular process	*q*-value
Regulation of activated PAK-2p34 by proteasome mediated degradation	Apoptosis	0.039
Regulation of Apoptosis	Apoptosis	0.042
DNA strand elongation	Cell cycle	0.023
FBXL7 down-regulates AURKA during mitotic entry and in early mitosis	Cell cycle	0.050
G1/S DNA Damage Checkpoints	Cell cycle	0.023
Orc1 removal from chromatin	Cell cycle	0.012
p53-Dependent G1 DNA Damage Response	Cell cycle	0.023
p53-Dependent G1/S DNA damage checkpoint	Cell cycle	0.023
p53-Independent DNA Damage Response	Cell cycle	0.043
p53-Independent G1/S DNA damage checkpoint	Cell cycle	0.043
SCF(Skp2)-mediated degradation of p27/p21	Cell cycle	0.023
Stabilization of p53	Cell cycle	0.047
Switching of origins to a post-replicative state	Cell cycle	0.034
Synthesis of DNA	Cell cycle	0.016
Transcriptional activation of cell cycle inhibitor p21	Cell cycle	0.047
Transcriptional activation of p53 responsive genes	Cell cycle	0.047
Ubiquitin Mediated Degradation of Phosphorylated Cdc25A	Cell cycle	0.043
The role of GTSE1 in G2/M progression after G2 checkpoint	Cell cycle	0.010
GSK3B and BTRC:CUL1-mediated-degradation of NFE2L2	Cellular response to chemical stress	0.046
Nuclear events mediated by NFE2L2	Cellular response to chemical stress	0.042
Metallothioneins bind metals	Cellular response to metal ions	0.007
Response to metal ions	Cellular response to metal ions	0.010
NIK-->noncanonical NF-kB signaling	Cytokine signaling	0.047
ER-Phagosome pathway	Immune system, adaptive	0.047
Dectin-1 mediated noncanonical NF-kB signaling	Immune system, innate	0.043
Glucose metabolism	Metabolism of carbohydrates	0.019
Glycolysis	Metabolism of carbohydrates	0.010
Cholesterol biosynthesis	Metabolism of lipids	0.019
Creatine metabolism	Metabolism of proteins	0.023
Striated Muscle Contraction	Muscle contraction	0.000
Complex I biogenesis	Respiratory electron transport	0.050
Respiratory electron transport	Respiratory electron transport	0.030
Respiratory electron transport, ATP synthesis by chemiosmotic coupling, and heat production by uncoupling proteins.	Respiratory electron transport	0.012
mRNA Splicing - Major Pathway	RNA metabolism	0.048
Processing of Capped Intron-Containing Pre-mRNA	RNA metabolism	0.031
rRNA modification in the nucleus and cytosol	RNA metabolism	0.047
Transport of Mature Transcript to Cytoplasm	RNA metabolism	0.047
AUF1 (hnRNP D0) binds and destabilizes mRNA	RNA metabolism	0.034
Hedgehog ligand biogenesis	Signal transduction	0.038
Negative regulation of NOTCH4 signaling	Signal transduction	0.036
Hh mutants abrogate ligand secretion	Signal transduction	0.039
Hh mutants are degraded by ERAD	Signal transduction	0.047
Degradation of AXIN	WNT signaling	0.035

b		
Branched-chain amino acid catabolism	Amino acid metabolism	0.031
Adherens junctions interactions	Cell junction organization	0.010
Cell-cell junction organization	Cell junction organization	0.023
Complement cascade	Complement cascade activation	0.010
Regulation of Complement cascade	Complement cascade activation	0.007
Defective B3GALTL causes PpS	Glycosaminoglycan metabolism	0.010
Tie2 Signaling	Hemostasis	0.038
O-glycosylation of TSR domain-containing proteins	Post-translational protein modification	0.012
SHC1 events in ERBB2 signaling	Signal transduction	0.047
RUNX1 regulates transcription of genes involved in BCR signaling	Transcription	0.040
RUNX1 regulates transcription of genes involved in interleukin signaling	Transcription	0.048
RUNX2 regulates genes involved in cell migration	Transcription	0.014

The *q*-value is the false discovery rate-adjusted *p*-value calculated by the Camera method (correlation adjusted mean rank) and describes the statistical significance of pathway regulation.

### Effects of dexamethasone and lithium on active β-catenin expression in metatarsals and the human growth plate

**Fig. 5:**
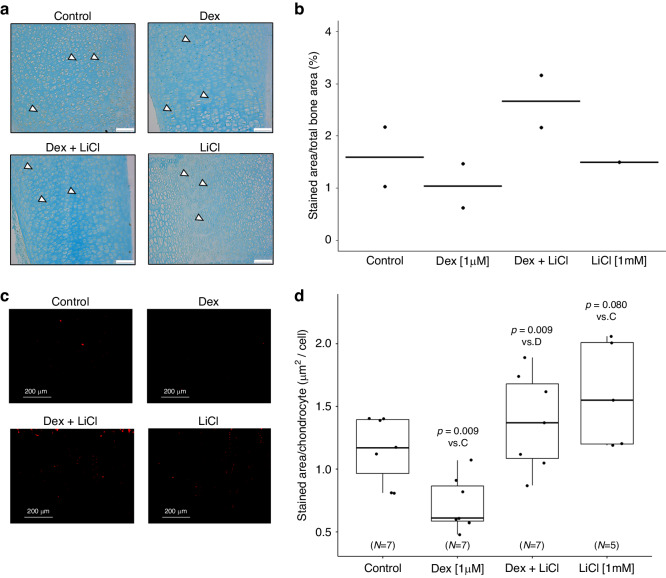
Active β-catenin expression in fetal rat metatarsals and human growth plate biopsies. LiCl prevented dexamethasone-induced decrease of active β-catenin. **a** Representative microscopic immunohistochemistry images of metatarsals (β-catenin stained brown, counterstained with Alcian blue; original magnification 20×, scale bar = 75 μm). Representative positive cells are marked with white arrows. **b** Quantification of the protein expression (dotplots with horizontal line representing the mean). **c** Representative microscopic immunofluorescent images of human growth plate biopsy samples (active β-catenin stained red; scale bar = 200 μm). **d** Quantification of the protein expression. Boxplots summarise the median (line inside the box), first and third quartile (i.e. Q1 and Q3, respectively; lower and upper box margin), minimum and maximum (Q1–1.5 × IQR and Q3 + 1.5 × IQR, where IQR means interquartile range; whiskers) and, eventually, outliers ( < Q1–1.5 × IQR or >Q3 + 1.5 × IQR). *P*-values were obtained as a result of Welch’s two-sample t-tests with FDR correction. Dex dexamethasone, LiCl lithium chloride.

The percentage of active-β-catenin-positively stained area within the total area of the metatarsal bone is presented in Fig. 5a, b. The total area of active-β-catenin-positively stained cells (in μm^2^) normalized to the number of cells within the same area of the human growth plate sections is shown in Fig. 5c, d. By adding dexamethasone to the culture medium, expression of β-catenin decreased compared to the control group, whereas LiCl did not change the expression. When dexamethasone was combined with LiCl, the β-catenin expression was significantly increased compared to the dexamethasone only group and was similar to the control group (Fig. 5d).

### Effects of dexamethasone and lithium on proliferation and apoptosis in metatarsals

**Fig. 6:**
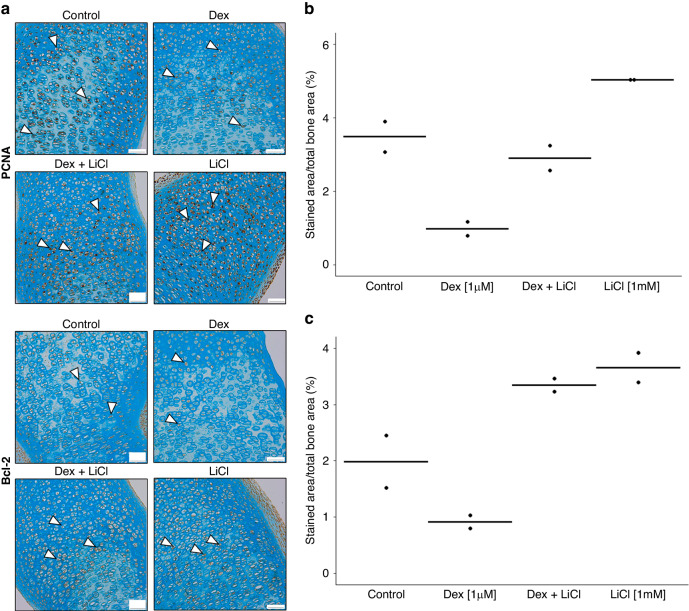
Expression of proliferation and antiapoptotic markers in fetal rat metatarsals. LiCl counteracts the dexamethasone-induced decrease of expression of PCNA and Bcl-2 in fetal rat metatarsals. **a** Representative microscopic immunohistochemistry images of metatarsals (PCNA and Bcl-2, respectively, stained brown, counterstained with Alcian blue; magnification 20×, scale bar = 50 μm). Representative positive cells are marked with white arrows. **b**, **c** Quantification of PCNA and Bcl-2 protein expression, respectively (dotplots with horizontal line representing the mean). PCNA proliferative cell nuclear antigen, Bcl-2 B-cell lymphoma 2 antigen, Dex dexamethasone, LiCl lithium chloride.

Whereas dexamethasone treatment caused decreased expression of a proliferation marker Proliferative cell nuclear antigen (PCNA) and an anti-apoptotic marker B-cell lymphoma 2 (BCL-2), as compared to control group, LiCl reverted these effects when added to dexamethasone (Fig. 6).

## Discussion

Our data showed that LiCl can partially rescue cultured fetal rat metatarsal bones from glucocorticoid-induced growth retardation and that, besides increased cell proliferation and energy production, these protective effects could be explained by the upregulation of Wnt and Hedgehog signaling pathways. In addition, LiCl restored dexamethasone-induced decrease of active β-catenin expression in human growth plate biopsies cultured ex vivo, which further supported the role of Wnt/ β-catenin signaling as one of the underlying molecular mechanisms.

The significant finding of this study was that LiCl can partially prevent glucocorticoid-induced suppression of fetal rat metatarsal bone growth. The effects of lithium on chondrocytes have been previously investigated in studies focusing on articular cartilage. In a surgical osteoarthritis in vivo model, LiCl provided both in drinking water and intra-articularly was shown to improve the osteoarthritis score and to reduce the severity of cartilage destruction.^25^ Interestingly, it has been demonstrated that when primary bovine and human chondrocytes were treated with the combination of LiCl and inflammatory cytokines (i.e. IL-1β, TNFα), LiCl showed the potential not only to downregulate proteins and genes related to cytokine signaling, but also to revert the deleterious effects of cytokines on extracellular matrix.^25–27^ In the longitudinal bone growth setting, the results obtained from our proof-of concept experiments are supported by a previous study where lithium carbonate administration increased the width of the proximal tibia growth plate in the domestic fowl.^28^ The growth-promoting effect of LiCl was only minor in our “healthy” fetal rat metatarsals, compared to the effect in dexamethasone-impaired metatarsals. A possible explanation to this finding is the naturally high growth rate of cultured fetal rat metatarsals that may disguise the proliferative potential of LiCl. However, this potential was present when bone growth was suppressed by dexamethasone. Notably, the cytoprotective properties of LiCl have been highlighted in numerous preclinical studies where lithium improved fracture healing,^29^ prevented the development of Parkinson disease,^30^ stimulated podocyte renewal in Adriamycin-induced glomerulonephritis.^31^ and protected neural progenitors from irradiation-induced damage.^32^ Taken together, these studies and our findings suggest that LiCl has the potential to exert reparative effects in different damaged tissues, including the glucocorticoid-stressed growth plate.

We explored the underlying molecular mechanisms of the protective effect of LiCl upon dexamethasone-induced growth retardation in fetal rat metatarsals by gene expression profiling. On the individual gene level, cell attachment, cytokine production, chemokine binding, G protein-coupled receptor signaling, transcription activity or cell kinesis were found to be affected. The gene set analysis of the RNA-seq data revealed that the metatarsal tissue (primarily composed of chondrocytes) showed upregulation of numerous pathways linked to cell renewal when comparing the LiCl plus dexamethasone group with the dexamethasone alone group. Our study is the only one so far to apply RNA-seq method, so direct comparison to previous studies was not possible. However, we identified 12 studies that reported data on the action of LiCl on chondrocytes, in which other methods such as regular extracellular matrix stainings, Western blot, qRT-PCR or targeted immunofluorescence and immunocytochemistry were used (Table 2). Two studies applied in vivo models where LiCl was shown to upregulate the assembly of primary cilia in chondrocytes^33^ and to reduce cartilage destruction in a surgical model of osteoarthritis.^25^ In the majority of studies, lithium treatment was shown to have positive effects on chondrocytes via upregulating the expression of markers related to the extracellular matrix, chondrocyte differentiation, primary cilia elongation, cell cycle and metabolism. Interestingly, LiCl treatment was also shown to reverse the cytokine-induced negative effects on chondrocytes by regulating pathways related to signal transduction, extracellular matrix and the immune system.^25,27,34^ Contradictory data have been shown in two studies where LiCl was used in very high concentration (20 mM) or was used in primary human chondrocytes derived from patients with osteoarthritis.^35,36^ Based on these data, LiCl treatment increased senescence, oxidative damage-stress, apoptosis and upregulated catabolic enzymes. These observations underline the importance of the LiCl concentration used in the different experimental conditions and the experimental material used, as the reported effects on osteoarthritic cartilage were variable.

**Table 2:** Review of selected mechanistic studies summarizing the effects of LiCl on chondrocytes.

Study, Year	Dose/ concentration, route of administration	Treatment duration	Experimental model	Method	Proteins	Genes	Function/Pathway involved	Down/up
**In vivo**								
Thompson, et al.^33^	60 mM/100 g ground food	9 mo	Male Wistar rats	IF	a-tubulin		Chondrocyte primary cilia assembly	Up
Minashima, et al.^25^	ia 10 mM weekly or in drinking water final 20 mM	8 wks	Surgical OA mouse model (male C57/BL6, 10-wk-old)	Safranin O staining and OARSI scoring			Cartilage destruction	Down
**Ex vivo- in vitro**								
Kawasaki, et al.^44^	8 mM	72 h	ATDC5 cells (prehypertrophic,hypertrophic)	RT-PCR		Col10, MMP-13, ALP	Extracellular matrix differentiation	Up
			Primary murine E18.5 costal chondrocytes	ICC	Col10 MMP-13 ALP		Extracellular matrix differentiation	
Hui et al.^27^	0.1–10 mM + IL-1 (0.5 ng/ml), IL-1 (0.2 ng/ml) + OSM (4 ng/ml) or TNF-a (10 ng/ml)	24 h-14 days	Bovine nasal cartilage chondrocytes	Enzyme activity assays	Collagens-collagen release		Extracellular matrix (catabolism)	Down^a^
			Primary human chondrocytes (OA patients)	RT-PCR, ELISA (conditioned medium)	MMP-1 MMP-13		Extracellular matrix (catabolism)	
				western blot	p- HSP27		p38 MAPK (signal transduction, inflammation)	
Ning et al.^45^	10 mM	48 h	Primary chondrocytes (8-wk-old rats)	IF and TUNEL	β-catenin col10 MMP-13		Extracellular matrix apoptosis	Up
				qRT-PCR		col10, MMP-13 β-catenin ADAMTS-5	Extracellular matrix differentiation	
Krase et al.^46^	10 mM	4 wks	Primary porcine articular chondrocytes cultured in collagen-type-I/III-matrices	qRT-PCR		axin2	Wnt-pathway signal transduction	up
Minashima et al.^25^	10 mM + IL-1β	5 days	Extracts of femoral head (18-wk-old mice)	DMMB assay western blot	GAG release ADAMTS-digested aggrecan fragments in culture medium		Extracellular matrix	Down^a^
			Human articular cartilage explants or chondrocytes	RT-PCR		Aggrecan, Col2a1	Extracellular matrix	Up^a^
						ADAMTS-5, COX-2, iNOS, and MMP-13,	Extracellular matrix gene expression (transcription) cellular responses to stimuli immune system	Down^a^
						IL-6	Cytokine signaling, immune system	
					IL-6 in culture medium		cytokine signaling, immune system	
		up to 24 h	Primary human articular chondrocytes	Western blot	p-p38		p38 MAPK (signal transduction)	
					p-STAT3		Immune system (IL-6, IL-1β/STAT3)	
Zhou et al.^34^	10 mM + IL-1β	24 h	Primary articular rabbit chondrocytes	Western blot	MMP-3 β-catenin		Signal transduction metabolism	Up^a^
					TIMP-1			Down^a^
				qRT-PCR		MMP-3 β-catenin		Up^a^
						TIMP-1		Down^a^
Guidoti et al.^36^	5 mM	up to 24 h	Primary human chondrocytes (OA patients)	SA- β-galactosidase staining PAS staining			Senescenceglycogenesis	Up
				ROS and mitochondrial staining			Oxidative damage-stress	
				Flow cytometry+light scattering analysis			Cell cycle Cells in S phase	
				western blot	γH2AX, p21, GADD45ß, IKKa		DNA damage response senescence differentiation	
				qPCR		IKKa MMP-10	Differentiation extracellular matrix	
Thompson et al.^33^	0–50 mM	up to 24 h	Primary bovine articular chondrocytes	ICC	Acelylated a-tubulin arl13b		Chondrocyte primary cilia elongation	Up
			Human articular chondrocytes					
Thompson et al.^26^	0–50 mM + IL-1β	up to 12 days	Primary bovine articular explants and chondrocytes	Griess assay	PEG2 and NO in culture medium		Metabolism inflammation immune system	Down^a^
				Western blot	iNOS, aggrecan		Extracellular matrix immune system	
				Hydroxyproline content DMMB assay	collagen and sGAG release in culture medium		Extracellular matrix	
Thompson et al.^47^	25 mM	24 h	Primary bovine articular chondrocytes (P0-P5)	ICC	acelylated a-tubulin arl13b		Primary cilia elongation	Up
Ding et al.^35^	20 mM	up to 72 h	Rat cartilage endplate chondrocytes(male SD rats, 12-wk-old)	SA- β-gal staining			Senescence	Up
				Annexin V-FITC and Propidium Iodide (Flow cytometry)			Apoptosis	
				RT-qPCR		MMP-13 ADAMTS-5	Catabolic enzymes	
						aggrecan	Extracellular matrix	Down
Soave et al.^48^	10–50 mM	24 h	P0-P2 articular chondrocytes of skeletally mature cattle (20-24 months)	IF	Acetylated α-tubulin		Primary cilia elongation	Up

*ALP* Alkaline phosphatase; *COX-2* cyclooxygenase 2, *DMMB* dimethylmethylene blue, *FITC* fluorescein isothiocyanate, *GAG* Glycosaminoglycan, *ICC* Immunocytochemistry, *IF* Immunofluorescence, *IL-1β* interleukin-1b, *iNOS* inducible nitric oxide synthase, ia intra-articular, *MMP* matrix metalloproteinase, *NO* nitric oxide, *OA* Osteoarthritis, *OARSI* Osteoarthritis Research Society International, *OSM* Oncostatin-M, *PGE2* prostaglandin E2, *SA-β‑gal* senescence‑associated β‑galactosidase, *SD* Sprague Dawley, *sGAG* sulfated glycosaminoglycan.

^a^LiCl rescued the cytokine-induced effects.

Our transcriptomic analysis showed that Wnt/β-catenin, Hedgehog and Notch4 signaling pathways, which are known to play key roles in cartilage development and regulate the function of chondrocytes,^37,38^ were upregulated in dexamethasone + LiCl group as compared to the dexamethasone alone group. We have previously shown that glucocorticoids trigger apoptosis in chondrocytes,^5^ leading to severe growth retardation both in ex vivo and in vivo models.^22,39^ However, the exact underlying mechanism was not clear. Interestingly, earlier reported in vitro data in another model system of cultured osteoblasts showed that glucocorticoids suppress osteoblast differentiation by activating the GSK3β enzyme^11^ and, on the contrary, that lithium can reverse the negative effect of glucocorticoids by inhibiting GSK3β.^7^ Moreover, lithium targets the Wnt/β-catenin pathway to increase the proliferation of human mesenchymal stem cells.^10^ As GSK3β is a known regulator of Wnt/β-catenin pathway and we observed that LiCl upregulates Axin degradation, which leads to β-catenin-induced transcriptional activity, Wnt/β-catenin pathway seems to be a plausible target that could explain the growth-promoting effect of LiCl in the dexamethasone-impaired metatarsals. We couldn’t verify this hypothesis by quantifying the active-β-catenin expression directly in our metatarsals due to limited tissue that was available. However, our findings in human growth plate samples, where the addition of LiCl to dexamethasone restored the active-β-catenin expression, which was decreased due to dexamethasone (when compared to control), further supports the suggestion that regulation of Wnt/β-catenin pathway might play an important role in preventing the toxic effects of dexamethasone in both rat and human chondrocytes. In addition, as lithium probably counteracted the decrease in proliferation and antiapoptosis (PCNA and Bcl-2 protein markers, respectively) in the metatarsals, targeting these two cellular processes seems also a promising approach. In contrary, there was only minor overlap of pathways identified by our experimental approach between dexamethasone to control and LiCl to control or dexamethasone + LiCl to dexamethasone alone groups. This may suggest that the molecular mechanisms driving the bone growth effects that are induced by either dexamethasone or LiCl may differ and don’t necesarilly have a common primary molecular target. Nevertheless, our gene set analysis results and previously published studies alltogether provide evidence that LiCl is capable to induce cell proliferation and renewal which may counteract the deleterious effects of dexamethasone on the metatarsals.

The strength of our study is the complex molecular mechanism-exploring approach utilizing whole tissue transcriptome analysis and an effort to verify the observed effects in a unique ex vivo model of human growth plate tissue culture. The potential limitation of our study is that we have not tested concentrations of LiCl above 10 mM in our experiments. One reason was that clinically relevant serum concentrations of LiCl in subjects with psychotic disease are well below this, i.e., 0.5–1.2 mM.^40^ Moreover, higher doses of LiCl have clinically been associated with undesired side effects such as tremor, dizziness, nausea, polyuria, weight gain, hypercalcemia or hypothyroidism, which therefore limits the use of high-dose LiCl treatment.^41^ Interestingly, intracellular lithium concentrations were shown to be higher than those in the serum^42^ and specific in vivo conditions, such as magnesium and ATP concentrations, significantly influence the potency of LiCl to reduce the GSK3β activity.^43^ Thus, lower concentrations of LiCl are likely needed to evoke similar biological effects when tested in in vivo when compared to ex vivo models. This remains to be elucidated in in vivo studies in disease models where bone growth is impaired. We also couldn’t dissect out whether the drug-induced transcriptomic changes originate from cartilage or its surrounding perichondrium. However, as perichondrium is a physiological part of the metatarsal and indispensable for the longitudinal bone growth, and we were interested in effects on the whole-organ level, our results can be considered relevant, despite the lack of knowledge on the amount of contribution to the whole-organ effect stemming from either the cartilage or the perichondrium.

In conclusion, the evidence from this proof-of-concept study indicates that LiCl has the potential to partially prevent dexamethasone-induced bone growth failure. The human growth plate chondrocyte model and the exploratory gene set analysis of metatarsal transcriptome demonstrated that the bone growth-rescuing effects of LiCl were associated with cell proliferation and renewal, including the well-known pathways such as Wnt/β-catenin, Hedgehog and Notch. These findings not only add to our understanding of the action of LiCl on chondrocytes but are also encouraging for the potential development of new treatment strategies to prevent glucocorticoid-induced growth failure using LiCl.

## Supplementary information

Supplementary Table 1

Supplementary Table 2

Supplementary Table 3

Supplementary Table 4

Supplementary Table 5

Supplementary Table 6

## Data Availability

Gene count tables and raw sequencing data were deposited at NCBI Gene Expression Omnibus (GEO), and Sequencing Reads Archive (SRA) databases under accession number GSE186104.
