# Supplementary material for: Lithium rescues cultured rat metatarsals from dexamethasone-induced growth failure
Source: Pediatr Res. 2024 Apr 29;96(4):952–63. doi: 10.1038/s41390-024-03192-6 (PMC11502490; doi:10.1038/s41390-024-03192-6)
Supplement: Supplementary file 5 — Supplementary Table 5 [file 41390_2024_3192_MOESM5_ESM.pdf]

Table 1. Gene sets (pathways) found to be significantly regulated in dexamethasone + lithium group compared to dexamethasone alone group.

Cellular Process	Pathway Regulation	q-value	Pathway Name and Enrichment Plot
Apoptosis	Up	0.039	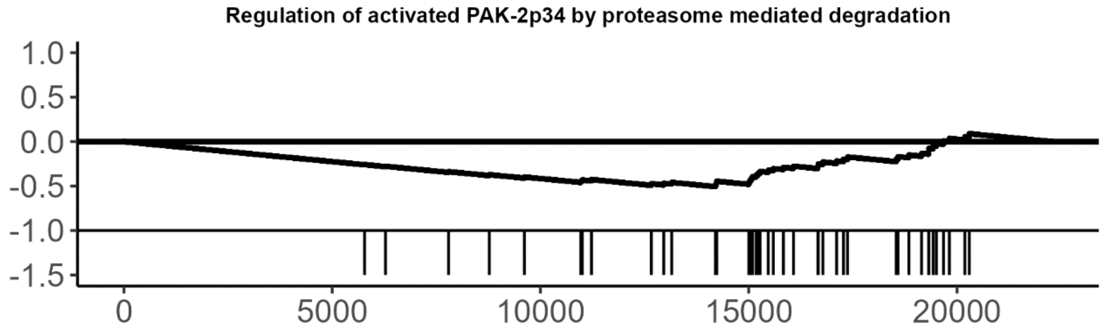
Apoptosis	Up	0.042	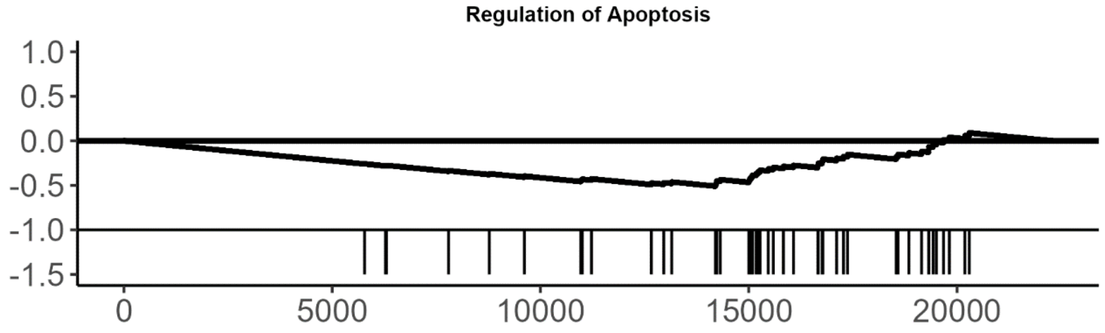

Amino acid metabolism	Down	0.031	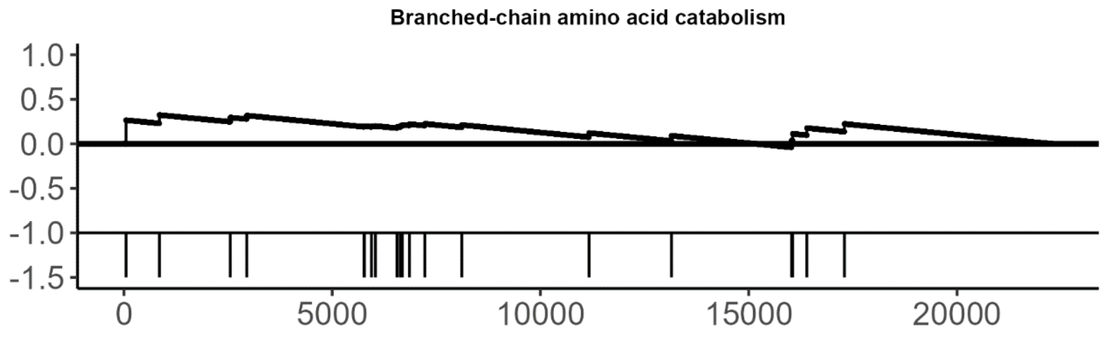
Cell cycle	Up	0.023	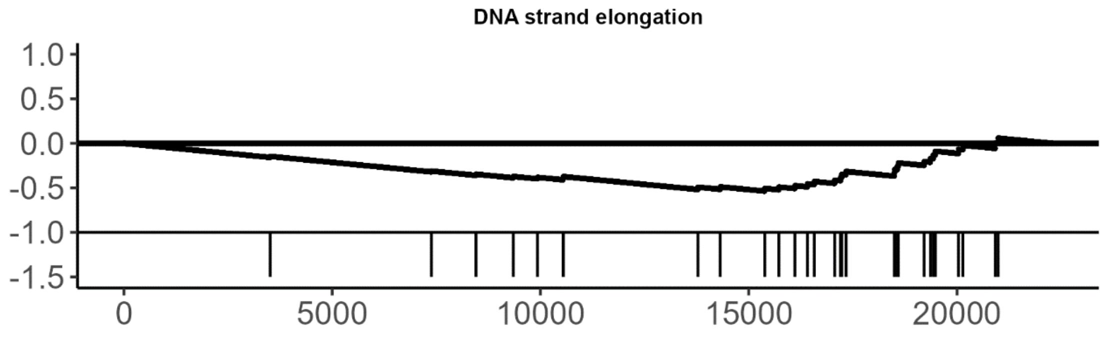
Cell cycle	Up	0.050	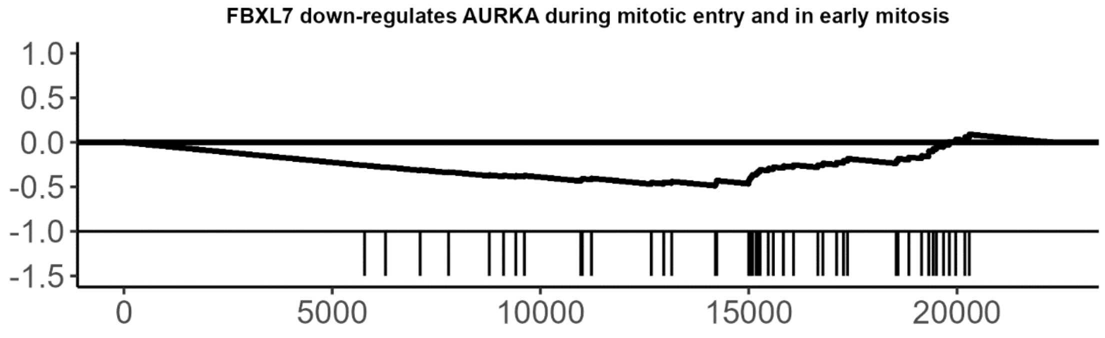

Cell cycle	Up	0.023	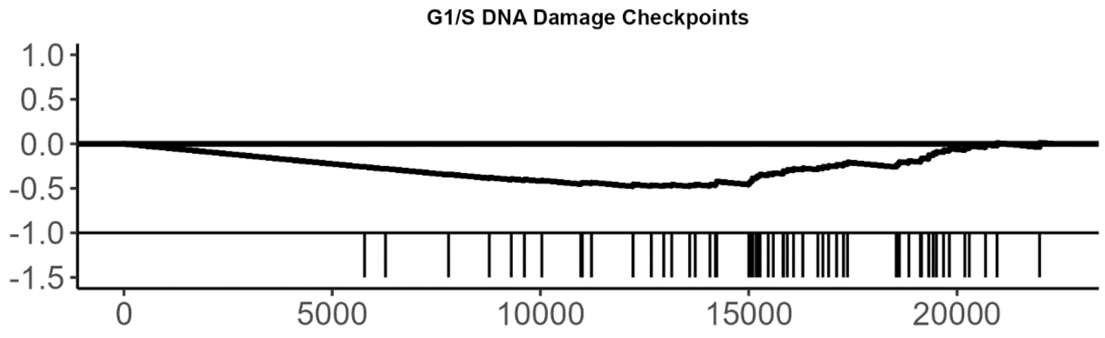
Cell cycle	Up	0.012	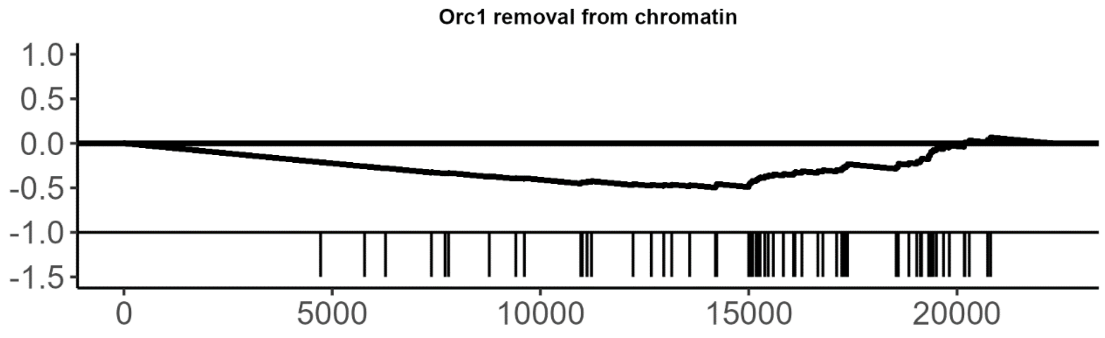
Cell cycle	Up	0.023	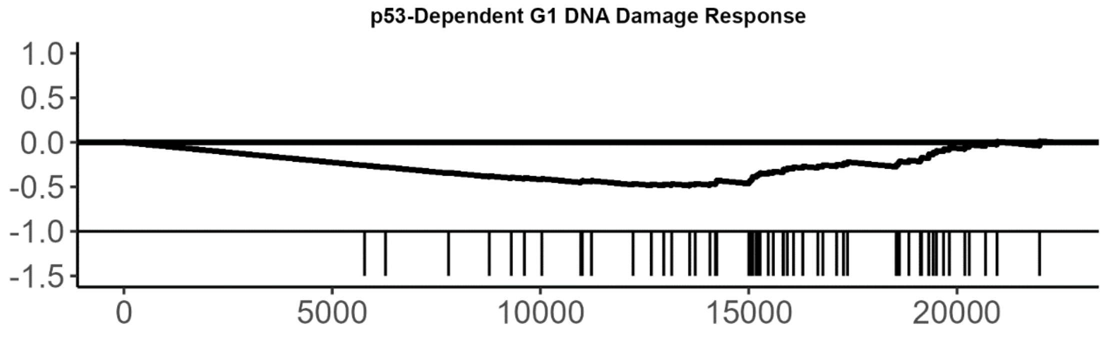

Cell cycle	Up	0.023	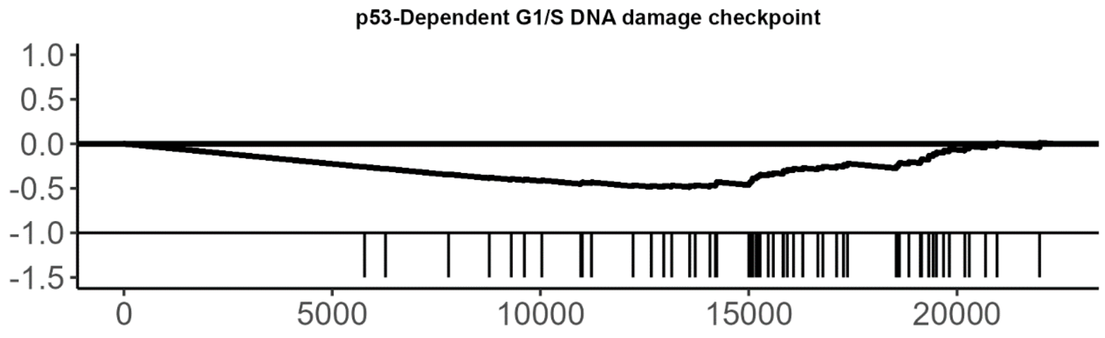
Cell cycle	Up	0.043	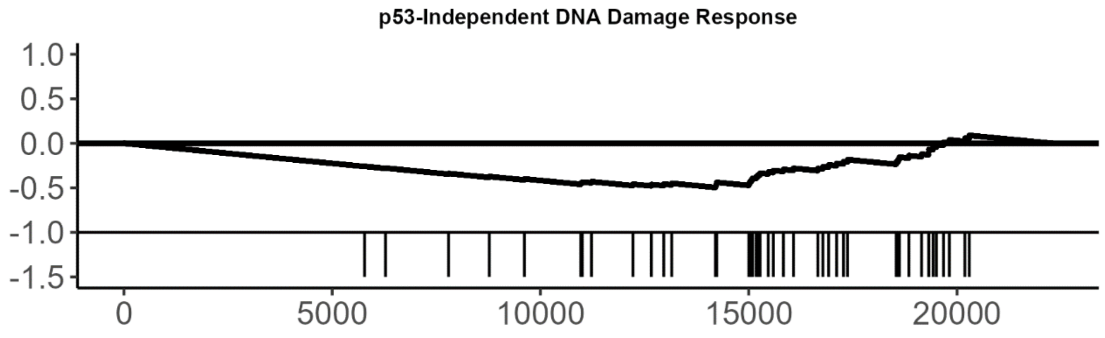
Cell cycle	Up	0.043	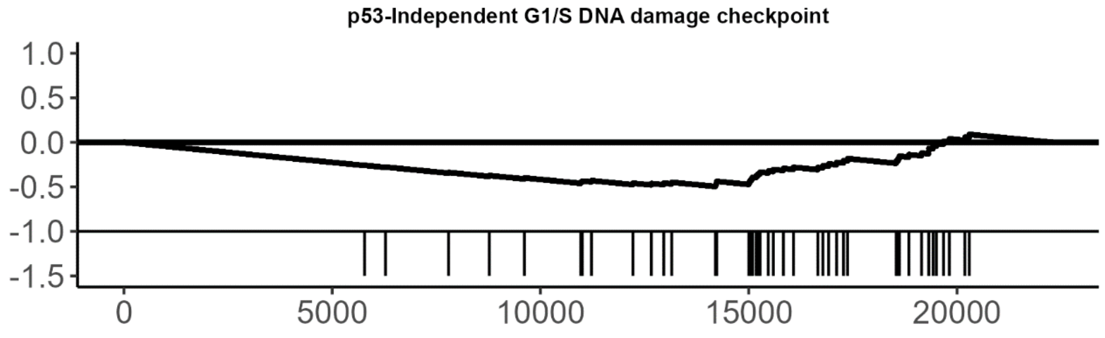

Cell cycle	Up	0.023	SCF(Skp2)-mediated degradation of p27/p21 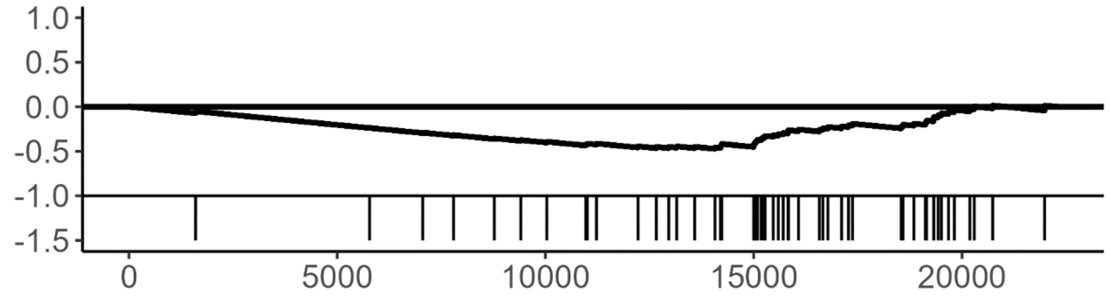
Cell cycle	Up	0.047	Stabilization of p53 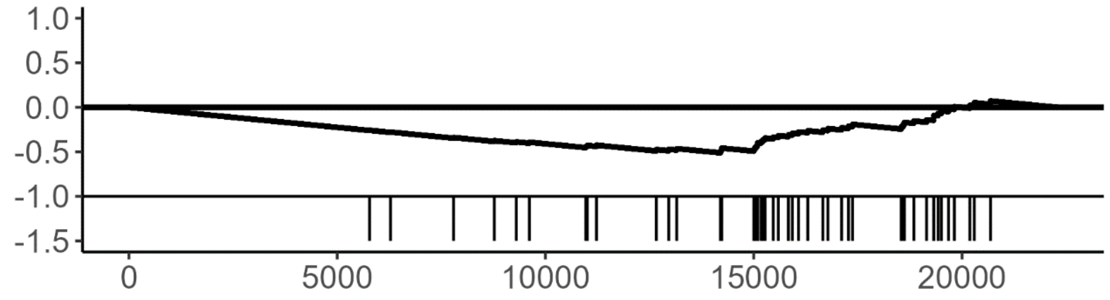
Cell cycle	Up	0.034	Switching of origins to a post-replicative state 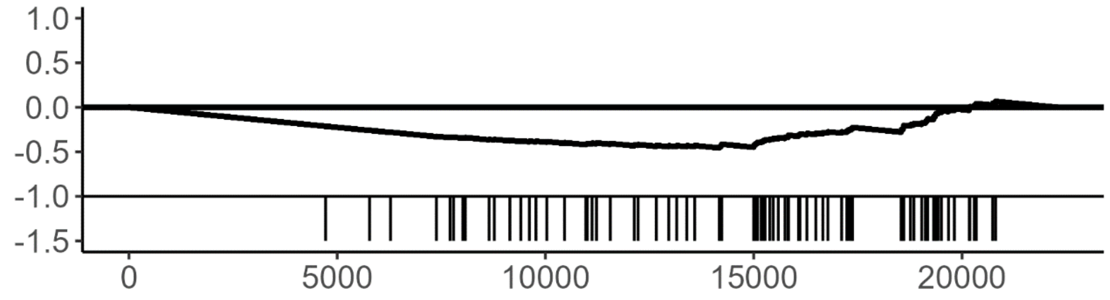

Cell cycle	Up	0.016	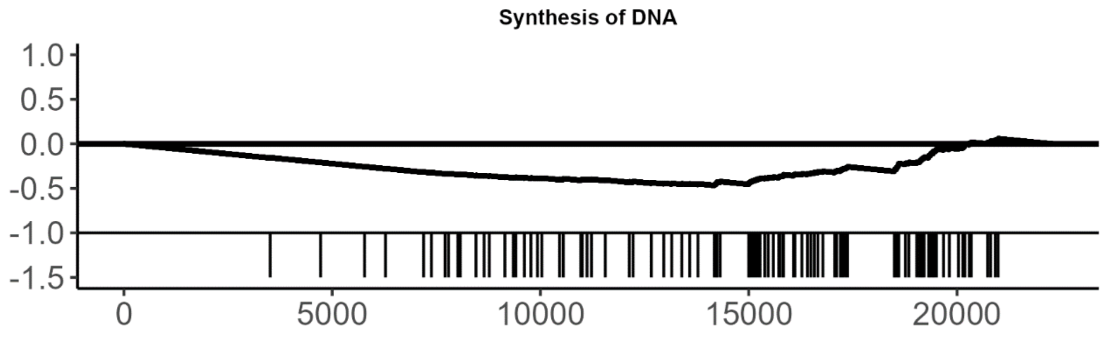
Cell cycle	Up	0.047	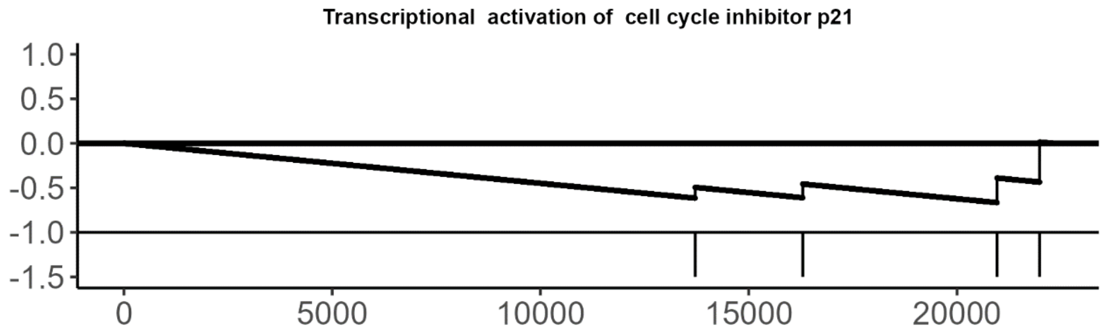
Cell cycle	Up	0.047	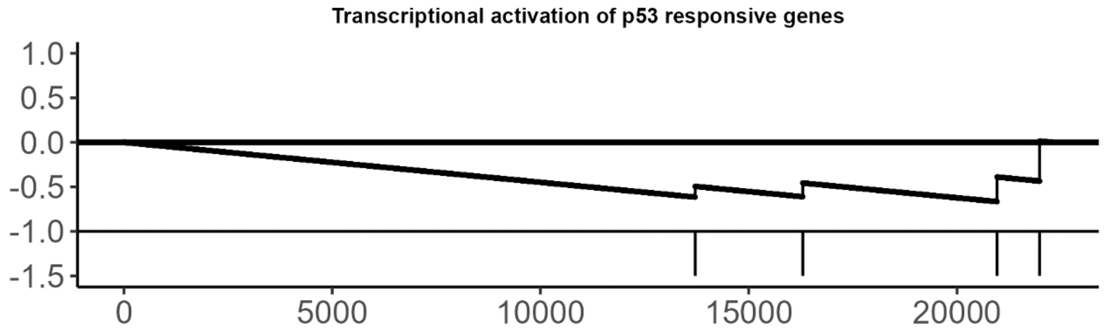

Cell cycle	Up	0.043	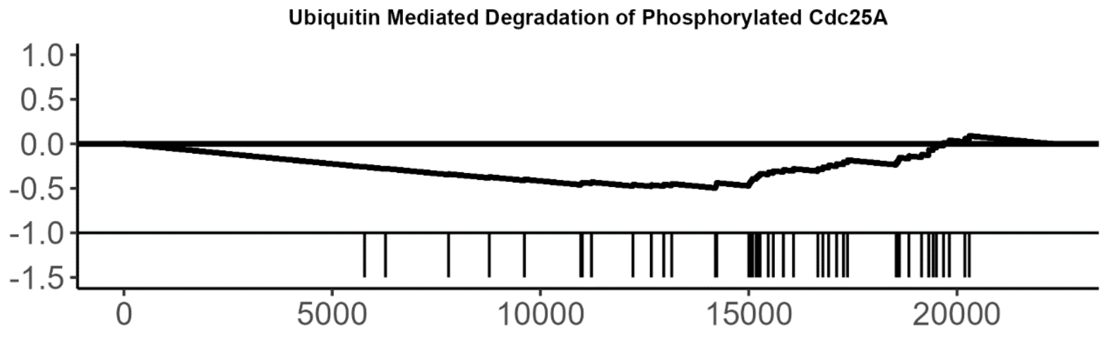
Cell cycle	Up	0.010	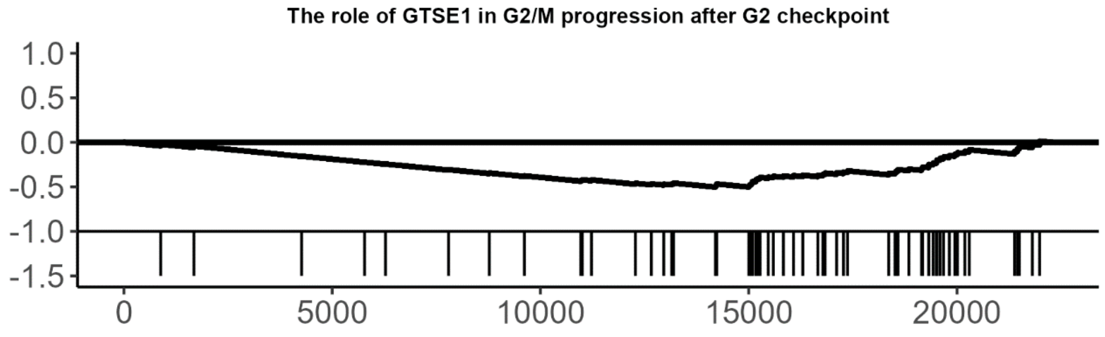
Cellular response to chemical stress	Up	0.046	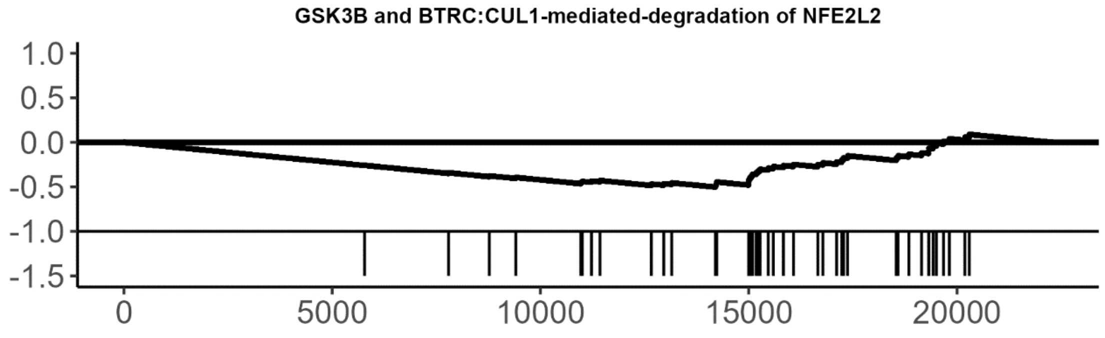

Cellular response to chemical stress	Up	0.042	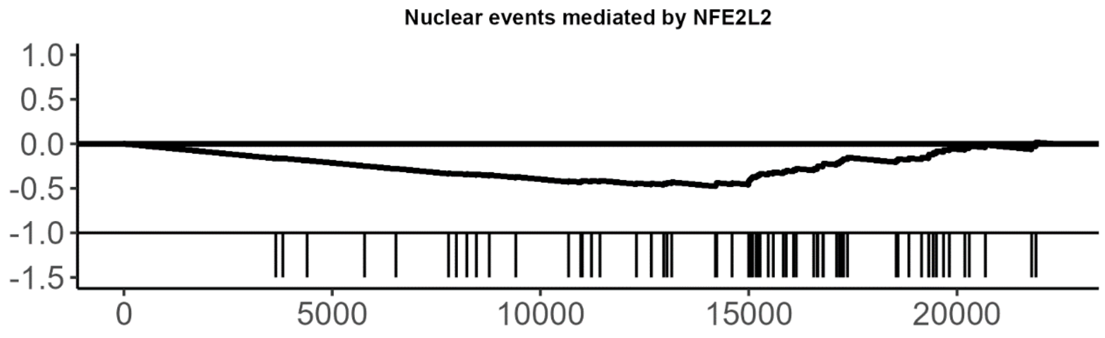
Cell junction organization	Down	0.010	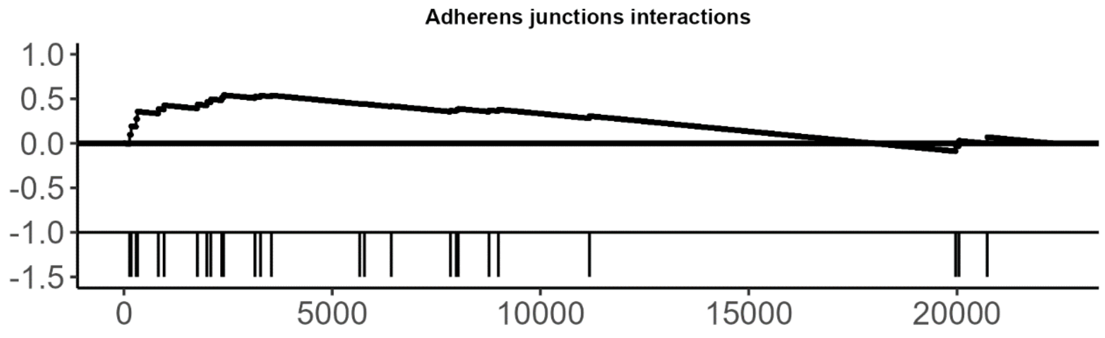
Cell junction organization	Down	0.023	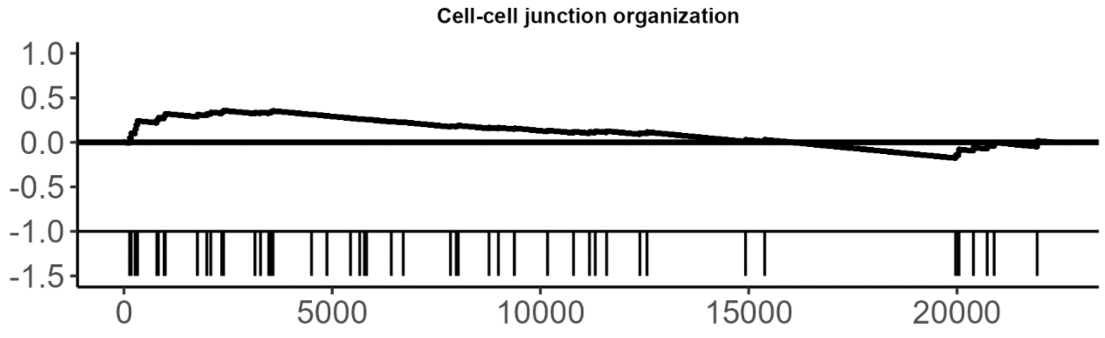

Cellular response to metal ions	Up	0.007	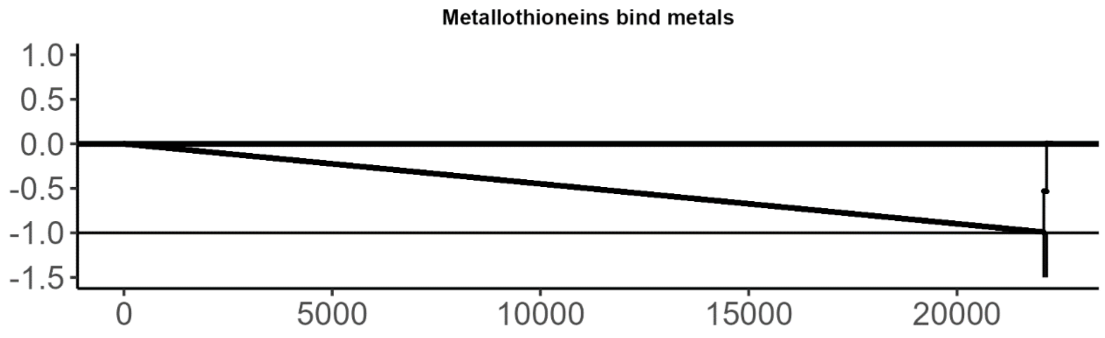
Cellular response to metal ions	Up	0.010	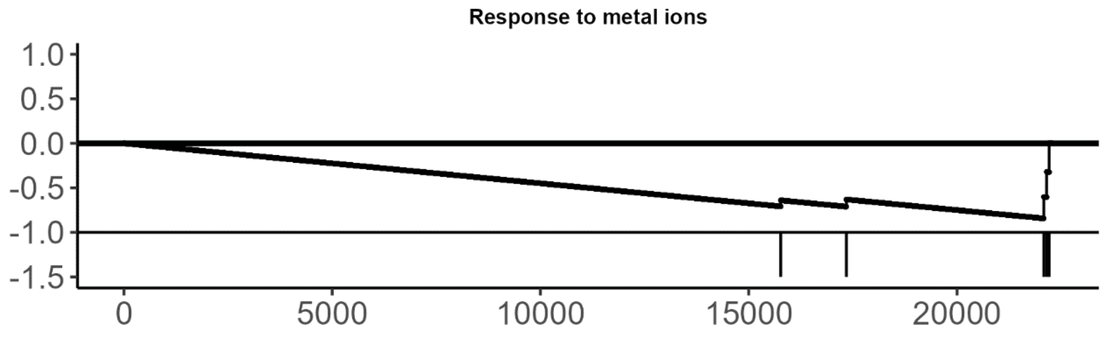
Cytokine signalling	Up	0.047	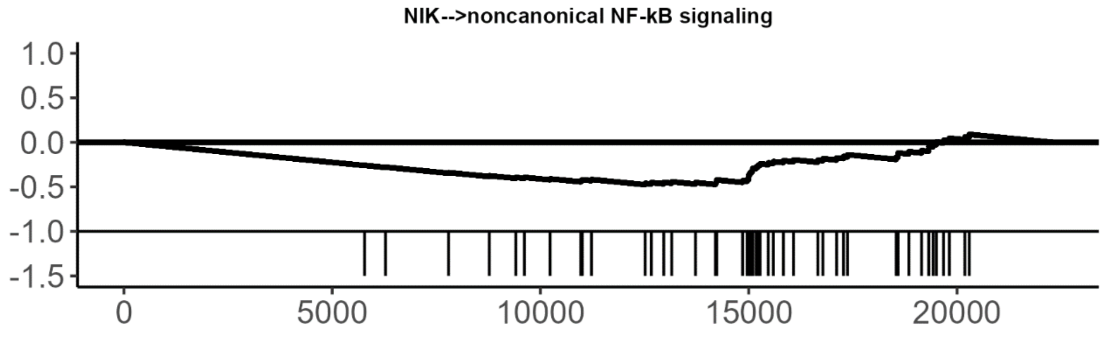

Complement cascade activation	Down	0.010	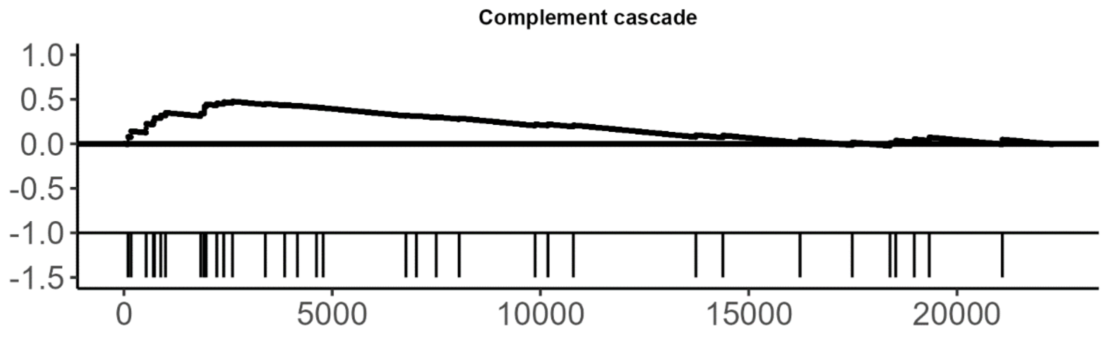
Complement cascade activation	Down	0.007	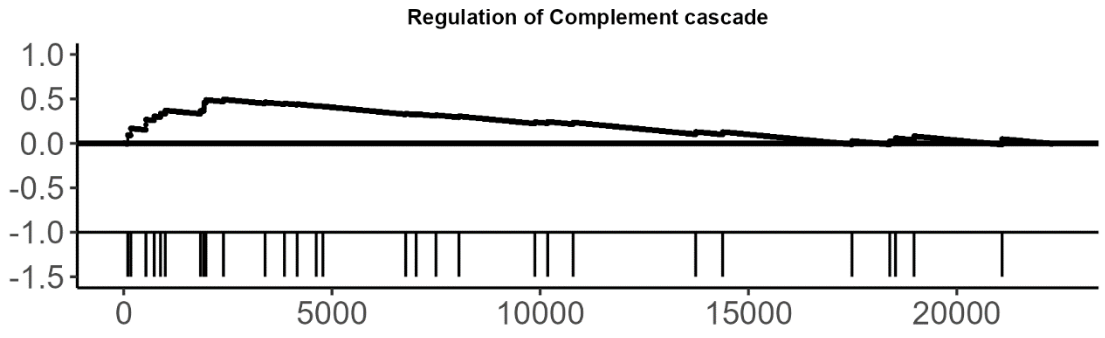
Glycosaminoglycan metabolism	Down	0.010	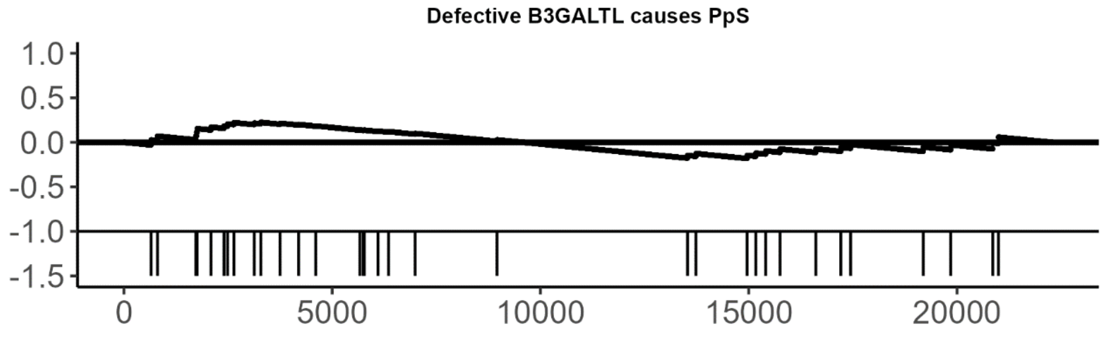

Immune system, adaptive	Up	0.047	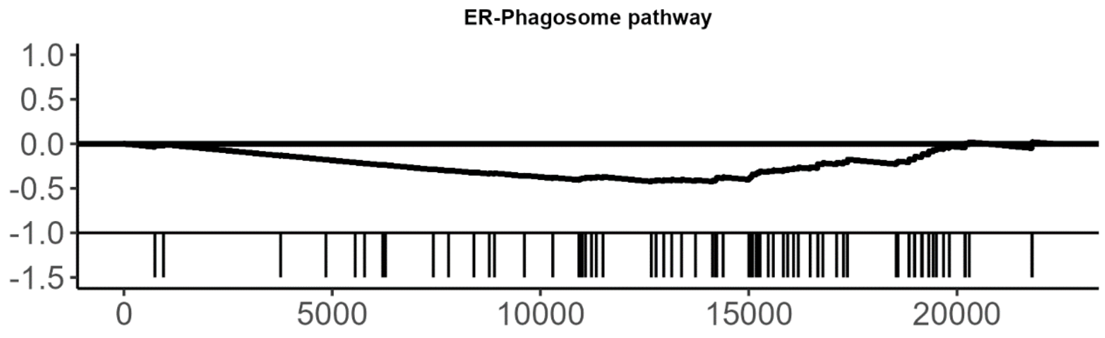
Immune system, innate	Up	0.043	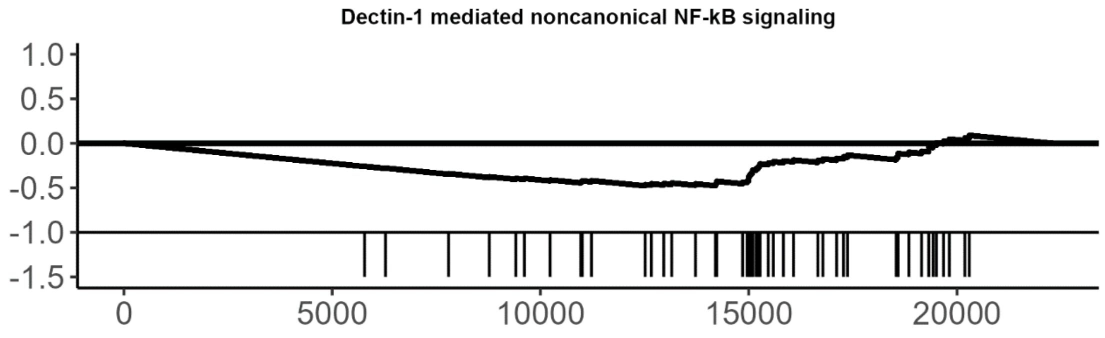
Metabolism of carbohydrates	Up	0.019	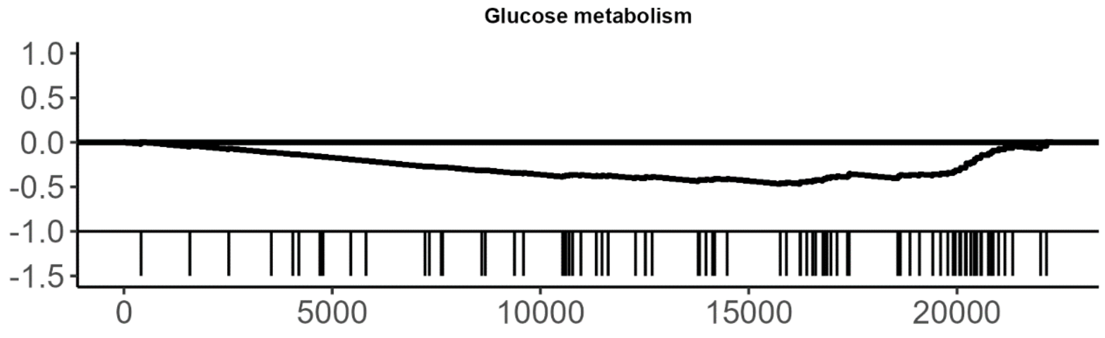

Metabolism of carbohydrates	Up	0.010	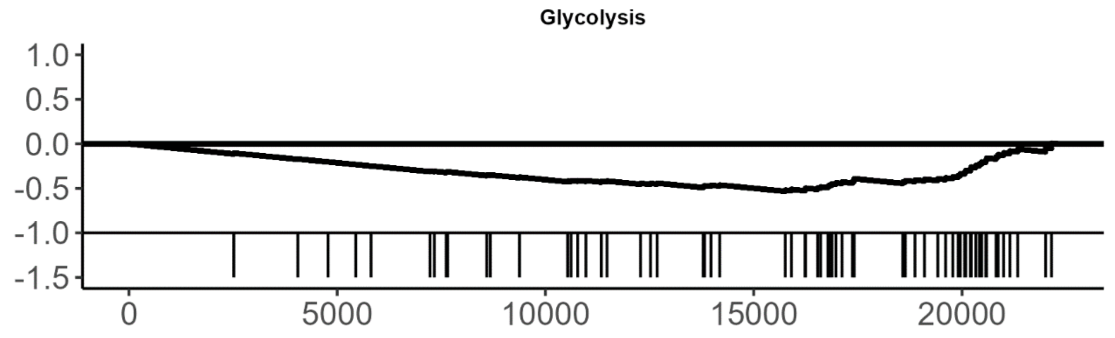
Hemostasis	Down	0.038	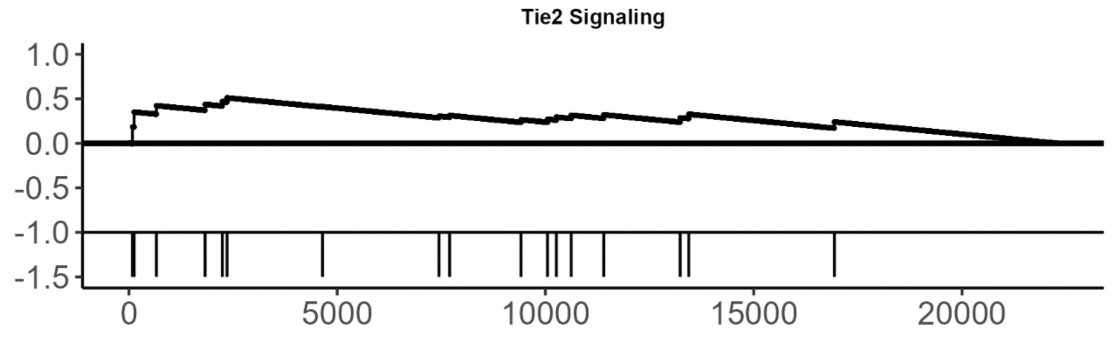
Metabolism of lipids	Up	0.019	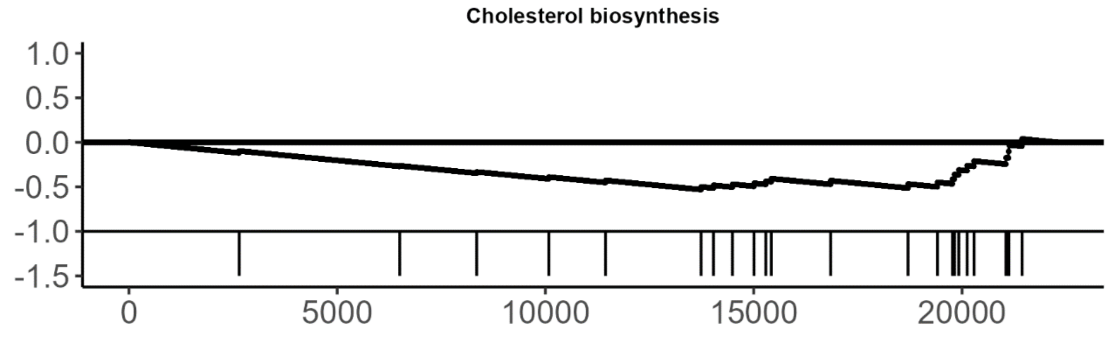

Metabolism of proteins	Up	0.023	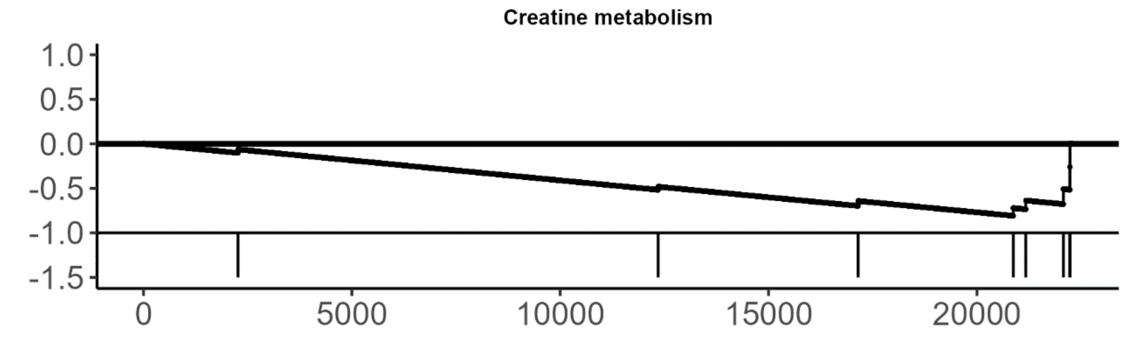
Muscle contraction	Up	0.000	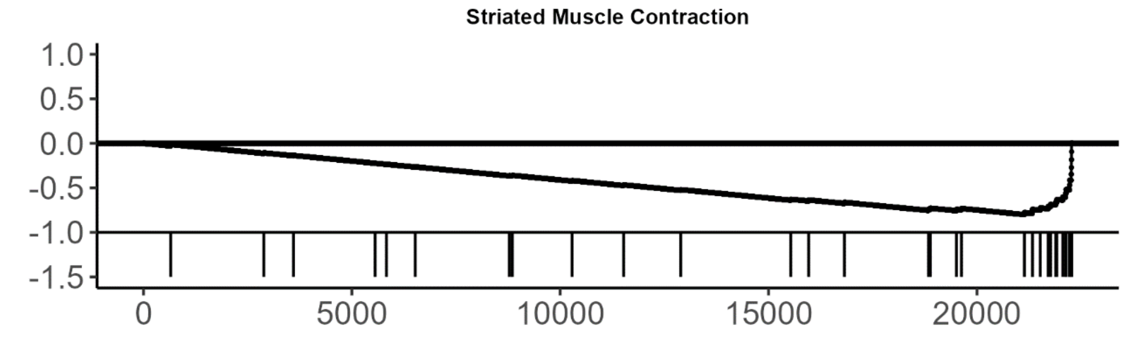
Respiratory electron transport	Up	0.050	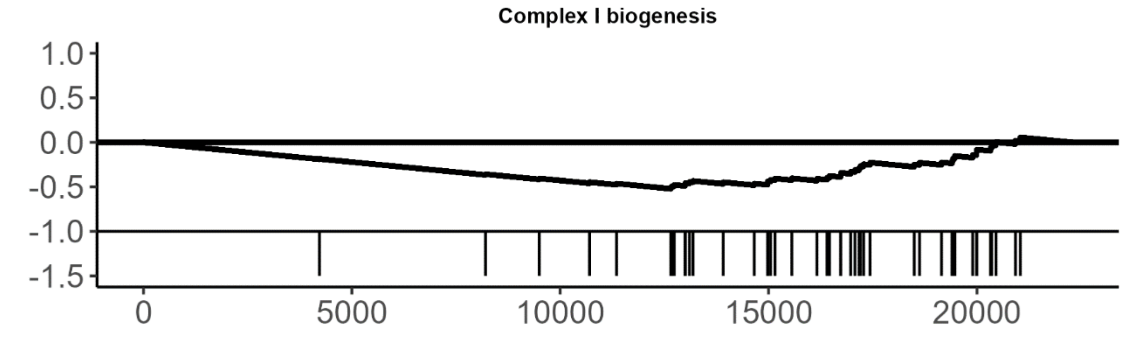

Respiratory electron transport	Up	0.030	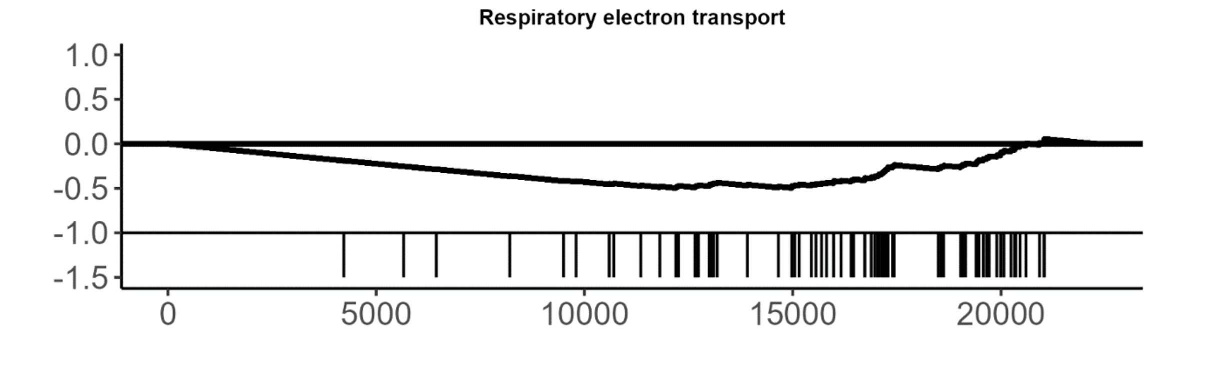
Post-translational protein modification	Down	0.012	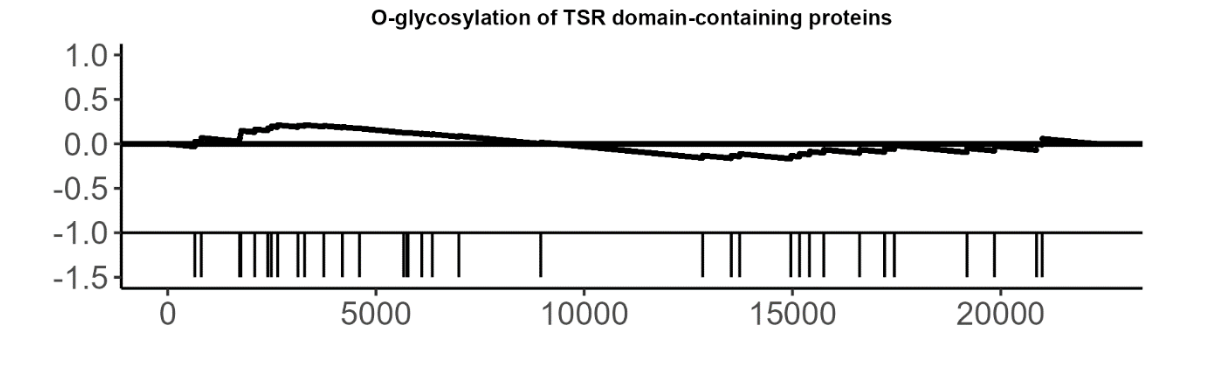
Respiratory electron transport	Up	0.012	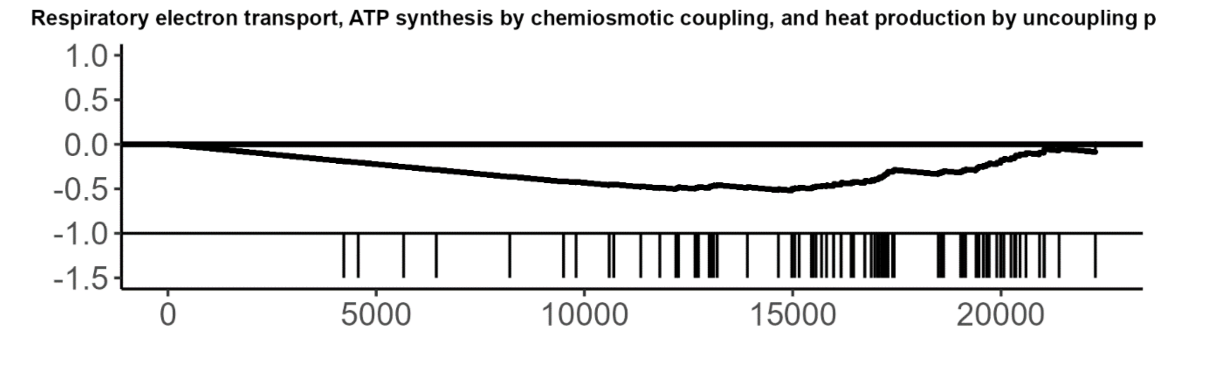

RNA metabolism	Up	0.048	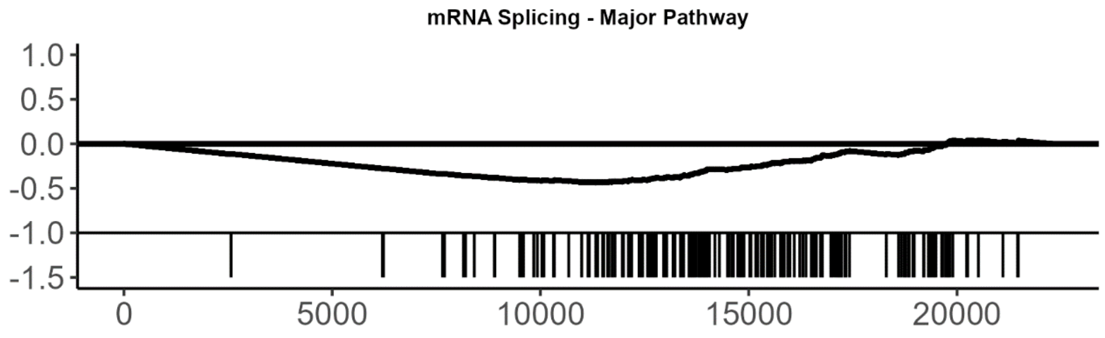
RNA metabolism	Up	0.031	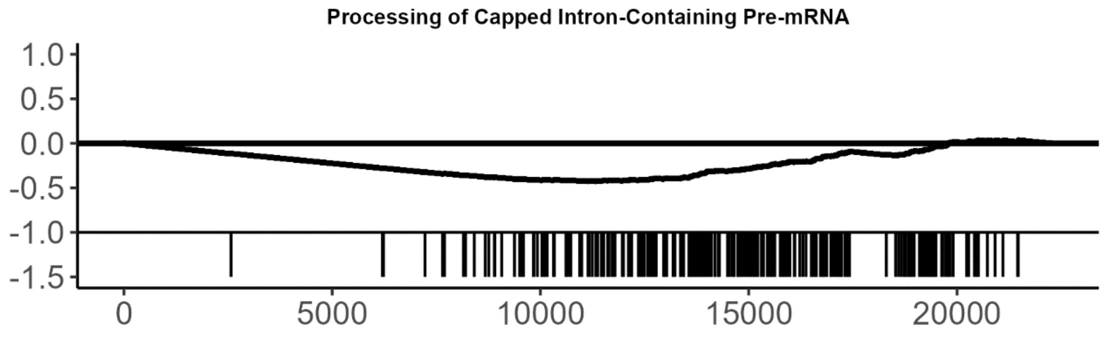
RNA metabolism	Up	0.047	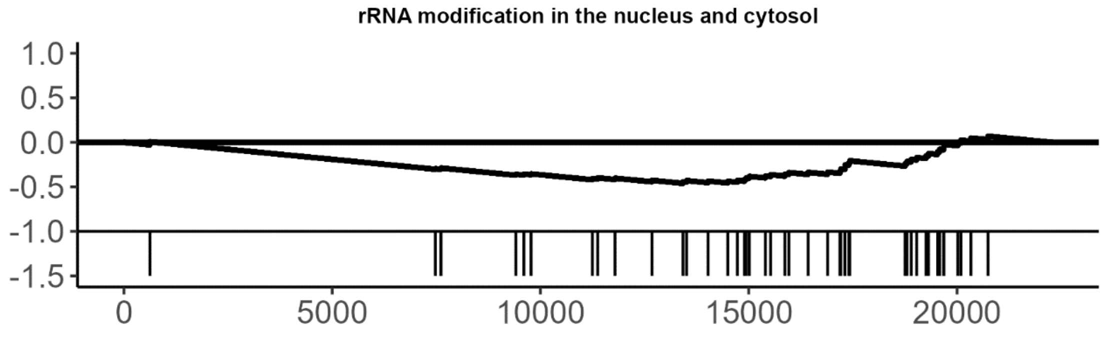

RNA metabolism	Up	0.047	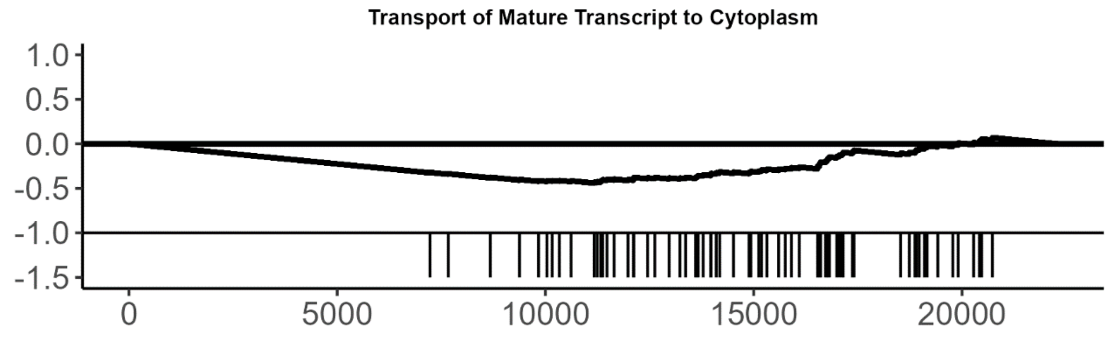
RNA metabolism	Up	0.034	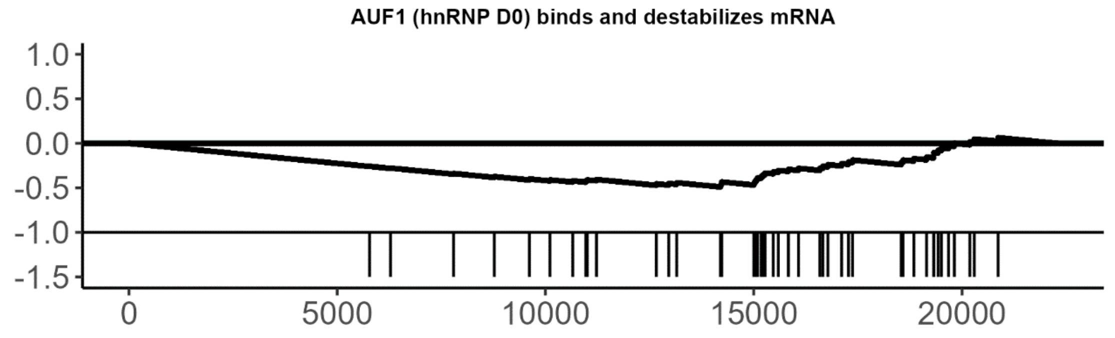
Signal transduction	Up	0.038	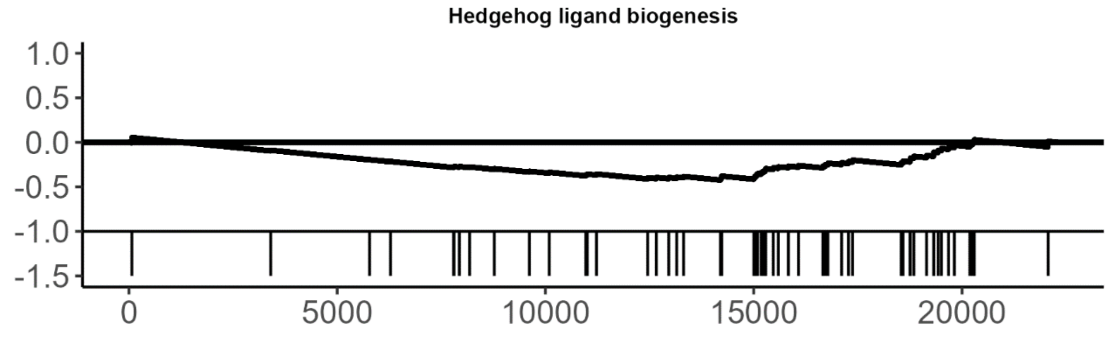

Signal transduction	Up	0.036	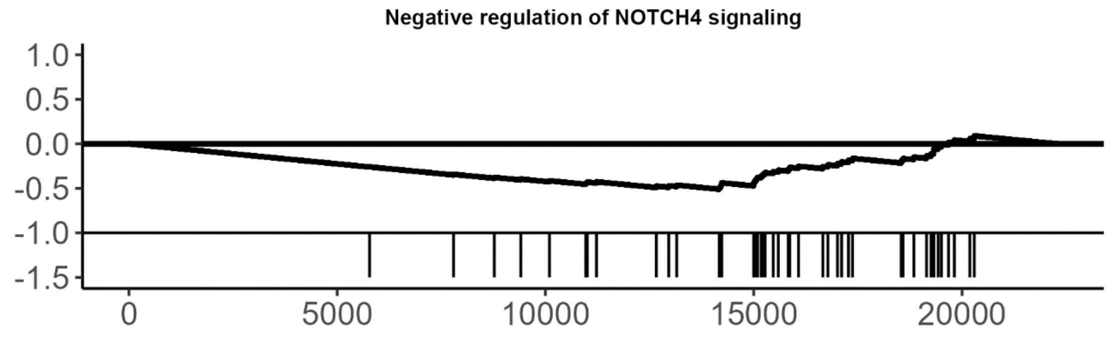
Signal transduction	Down	0.047	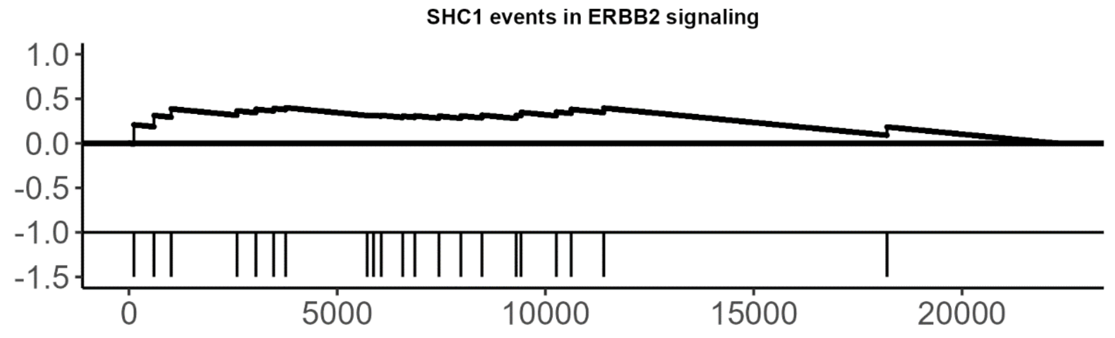
Signal transduction	Up	0.039	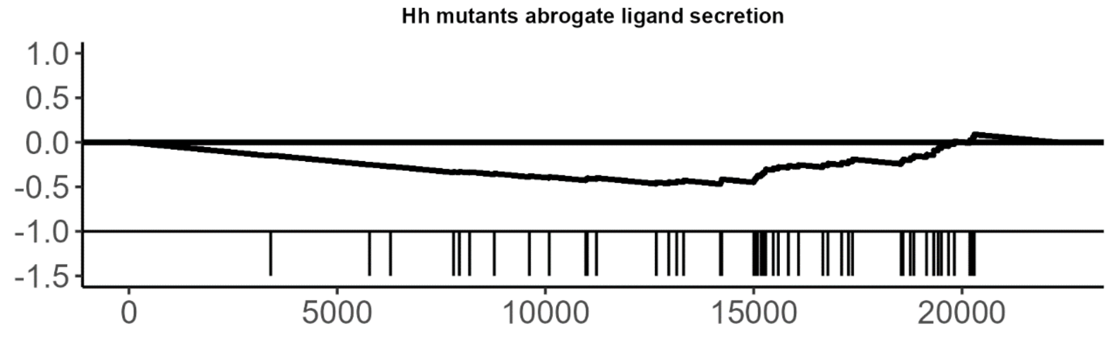

Signal transduction	Up	0.047	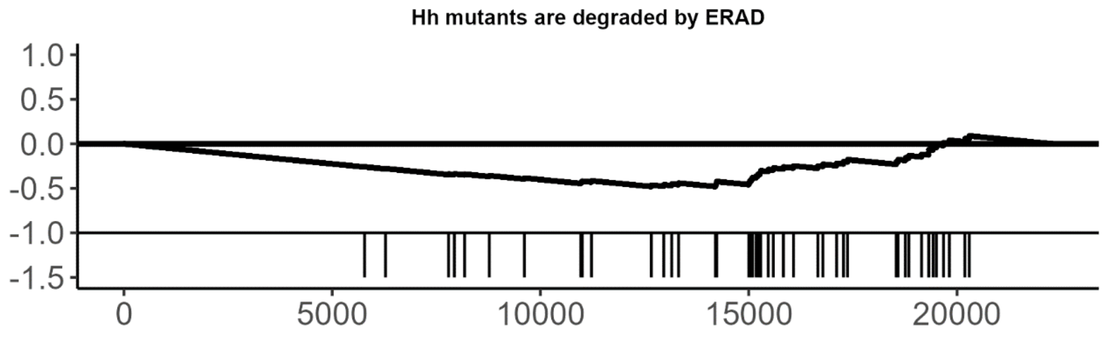
Transcription	Down	0.040	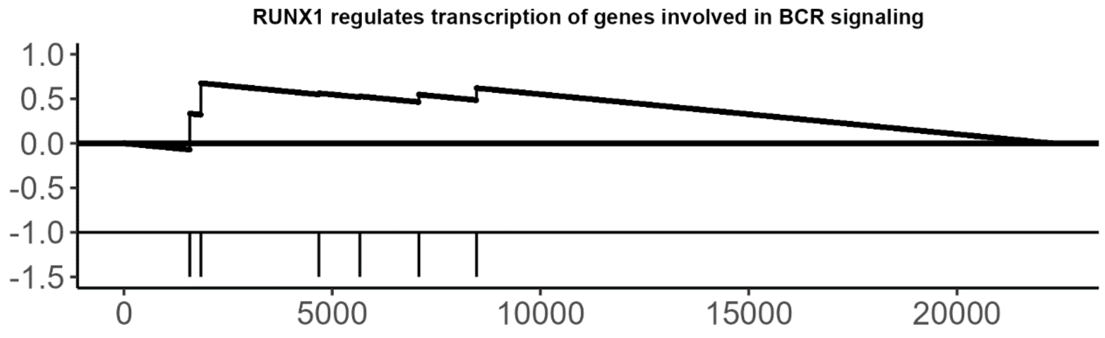
Transcription	Down	0.048	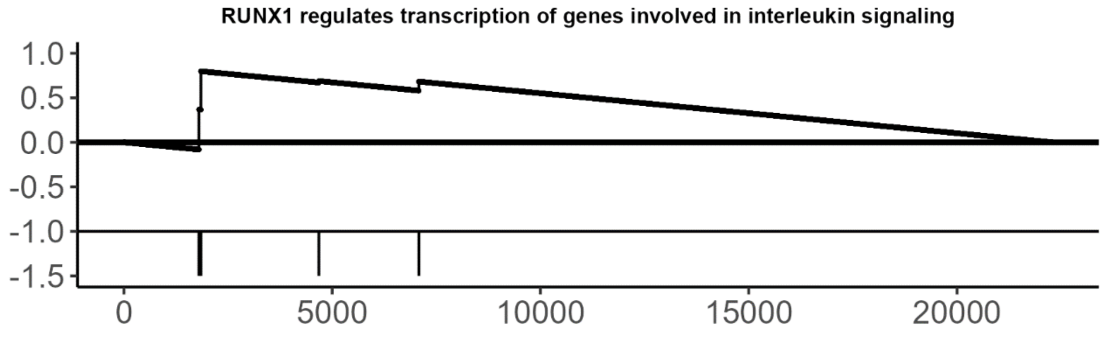

Transcription	Down	0.014	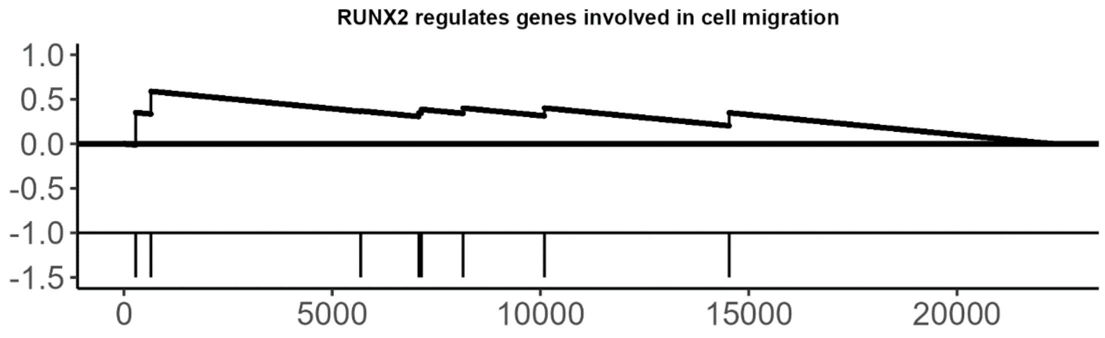
WNT signalling	Up	0.035	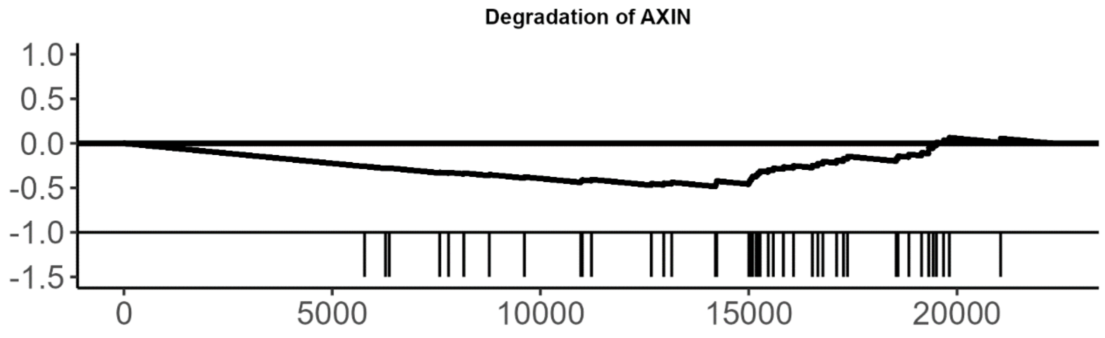

The q-value is the false discovery rate-adjusted p-value calculated by the Camera method (correlation adjusted mean rank) and describes the statistical significance of pathway regulation. Enrichment plots are based on basic gene set enrichment analysis (Kolmogorov Smirnov statistics).
